# Luminal Stem-like Cells in High-Risk/Locally Advanced Prostate Cancer Promote Resistance to Hormonal Therapy

**DOI:** 10.7150/thno.112496

**Published:** 2025-08-30

**Authors:** Guyu Tang, Jing Liu, Xiaomei Gao, Kai Yuan, Minfeng Chen, Long Wang, Lin Qi, Yi Cai

**Affiliations:** 1 Department of Urology, the Third Xiangya Hospital, Central South University; Postdoctoral Station of Basic Medicine, the Third Xiangya Hospital, Central South University, Changsha, China; Center for Experimental Medicine, the Third Xiangya Hospital, Central South University; Disease Genome Research Center, Central South University, Changsha City, 410013, Hunan Province, P.R. China.; 2 Department of PET/CT Center, Hunan Cancer Hospital/The Affiliated Cancer Hospital of Xiangya School of Medicine, Central South University, No.283 Tongzipo Road, Changsha City, 410013, Hunan Province, P.R. China.; 3 Hunan Key Laboratory of Molecular Precision Medicine, Department of Oncology, Xiangya Hospital, Central South University, No.87 Xiangya Road, Changsha City, 410008, Hunan Province, P.R. China.; 4 Department of Pathology, Xiangya Hospital of Central South University, No.87 Xiangya Road, Changsha City, 410008, Hunan Province, P.R. China.; 5 Department of Urology, Disorders of Prostate Cancer Multidisciplinary Team, National Clinical Research Center for Geriatric Disorders, Xiangya Hospital, Central South University, No.87 Xiangya Road, Changsha City, 410008, Hunan Province, P.R. China.

**Keywords:** high-risk/locally advanced prostate cancer, hormonal therapy, patient-derived organoids, single-cell RNA sequencing, stem-like cells, tumor microenvironment

## Abstract

**Introduction:** High-risk/locally advanced prostate cancer (HRLPC) accounts for a large proportion of prostate cancer cases in China and is associated with a high recurrence rate. Androgen deprivation therapy-based treatment offers limited benefits, which may be associated with changes in epithelial cells and the tumor microenvironment (TME) after treatment. However, the cellular composition and molecular characteristics of the subpopulations following hormonal treatment in HRLPC remain unclear.

**Methods:** To investigate the molecular characteristics of residual tumor samples in HRLPC patients following hormonal therapy and to identify the reasons for their high recurrence rates, this study performed single-cell sequencing on nine HRLPC patients. Additionally, by establishing patient-derived organoids (PDOs) and conducting drug screening, we analyzed epithelial cell subpopulations at different treatment stages and explored potential therapeutic strategies.

**Results:** This study identified a population of luminal stem-like epithelial cells (Lum stem-like) with high transcriptional activity of SOX9. After hormonal therapy, these cells were still alive and became the predominant component of epithelial luminal cells. Additionally, after hormonal therapy, the proportion of stromal components, such as fibroblasts and endothelial cells, significantly increased in the TME, and the intercellular communication between fibroblasts and other cells was enhanced. The level of immune infiltration decreased, but the proportion of FOXP3^+^ Treg cells increased, leading to an “exhausted” immune microenvironment state. We confirmed that PDOs can accurately reflect the epithelial subtypes of the primary tumor, such as Lum stem-like cells. Using 18 potential therapeutic agents at the organoid level for drug screening, the results showed that the Lum stem-like cells exhibited greater sensitivity to platinum-based drugs.

**Conclusions:** This study identified the dominant Lum stem-like epithelial cell subpopulation, along with changes in the TME characterized by increased stroma and decreased immune infiltration after hormonal therapy in HRLPC. These findings can help guide the subsequent treatment strategies for HRLPC patients.

## Introduction

Localized prostate cancer can be classified into localized low-and intermediate-risk prostate cancer, high-risk localized prostate cancer (Gleason score > 7 and/or prostate-specific antigen (PSA) > 20 ng/mL and/or clinical stage T3/T4), and locally advanced prostate cancer (any T and clinical N1) 1, 2. Localized low- and intermediate-risk prostate cancer has a high proportion in Europe and the United States and is associated with a favorable prognosis 3. However, high-risk/locally advanced prostate cancer (HRLPC) accounts for more than 50% of newly diagnosed prostate cancer cases in China 4-6. In addition, HRLPC accounts for two-thirds of prostate cancer-specific deaths within ten years 7. Androgen deprivation therapy (ADT) combined with potent androgen receptor (AR) signaling pathway inhibitors (such as enzalutamide) or androgen synthesis inhibitors (such as abiraterone) still cannot prevent HRLPC from progressing to castration-resistant prostate cancer (CRPC) due to resistance to hormonal therapy 8-11. The poor efficacy and unfavorable prognosis may be closely related to changes in tumor cells and the tumor microenvironment (TME), as well as intercellular interactions, under the pressure of hormonal therapy.

A large amount of single-cell transcriptomic data has been generated to characterize the tumor epithelial cells and TME in both newly diagnosed treatment-naïve localized prostate cancer and CRPC. Ren et al. performed single-cell sequencing on 12 radical prostatectomy (RP) samples from patients with newly diagnosed localized prostate cancer, revealing multiple transcriptional programs related to metastasis in primary prostate cancer 12. Other studies have analyzed CRPC, identifying its luminal cell lineage origins and transcriptional driving factors 13, 14. However, the composition of tumor cell subpopulations and intercellular interactions within the TME under hormonal therapy pressure in HRLPC, which is in an intermediate evolutionary state, remain unclear.

To identify the reasons for poor therapeutic efficacy in HRLPC patients and to analyze the dynamic changes in tumor cells and the TME following hormonal therapy, this study included nine HRLPC patients (two newly diagnosed and untreated, seven after hormonal therapy) for single-cell sequencing. We discovered and identified a group of luminal stem-like epithelial cells (Lum stem-like) that became the predominant component of epithelial cells after hormonal therapy. This subpopulation was insensitive to hormonal therapy and exhibited high transcriptional activity of stemness-related transcription factor SOX9. Furthermore, after treatment, there was a significant increase in the proportion of stroma, such as fibroblasts and endothelial cells (ECs) in the TME. The characteristic scoring of stromal cells like CXCL14^+^ fibroblasts was associated with poor prognosis. Communication between fibroblasts, Lum stem-like cells, and ECs was also enhanced after hormonal therapy. On the other hand, immune cell analysis revealed a significant decrease in the infiltration of CD4^+^ T cells, CD8^+^ T cells, and NK cells after treatment, and the immune microenvironment exhibited an “exhausted” state. To explore potential treatment strategies for HRLPC patients, we constructed patient-derived organoids (PDOs) from different stages of the patients before and after hormonal therapy. The results showed that PDOs faithfully reflected the luminal epithelial subpopulations of the primary tumor, such as Lum stem-like cells and neuroendocrine (NE) epithelial cells. We conducted drug sensitivity testing on 18 potential prostate cancer treatment-targeted drugs at the organoid level, which revealed that the Lum stem-like cell subpopulation was more sensitive to platinum-based drugs. This study provides valuable resources for revealing changes in the tumor epithelial composition and TME in HRLPC patients after hormonal therapy and guiding subsequent treatment directions.

## Methods

### Ethics declarations—ethics approval and consent to participate

All studies were conducted in compliance with relevant ethical guidelines. Research protocols involving human samples were reviewed and approved by the Ethics Committee of Xiangya Hospital, Central South University (202212810). Informed consent was obtained from all patients in accordance with the principles of the Declaration of Helsinki. Detailed clinicopathological information of the patients was provided in Supplementary Table S1 and S2.

### Sample collection and preparation

Nine patients diagnosed with prostate cancer through prostate needle biopsy and meeting the HRLPC criteria were included in this study. Seven patients received hormonal therapy with ADT plus AR signaling inhibitors before surgery. All patients underwent RP in Xiangya Hospital, Central South University. Prostate cancer tissue samples were collected based on preoperative imaging examination, needle biopsy reports, and macroscopic gross view. After sampling, tissues were immediately placed in tissue preservation medium (DMEM supplemented with 1% penicillin-streptomycin (Pen-Strep) (BioConcept, 4-01F00-H), 10 μM Y-27632 (Millipore, SCM075), and 100 µg/mL Primocin (InvivoGen, ant-pm-05)) and rapidly transported on ice to the laboratory. All tissue samples were divided into four portions on ice for single-cell RNA sequencing (scRNA-seq), organoid culture, formalin fixation and flash freezing in liquid nitrogen for storage.

### Isolation and culture of PDOs

PDOs were established based on previously reported protocols for prostate cancer organoid culture 15, 16 (Table S3). Briefly, tissue samples were washed with pre-chilled PBS and minced into small fragments approximately 1-2 mm³ in size, followed by digestion in adDMEM/F12 (Gibco, 12634010) containing 5 mg/mL Collagenase type II (Gibco, 17101015), 1% Pen-Strep and 10 μM Y-27632 on a shaker at 37°C for 2-5 h. The fragments were pipetted every 30 min until they were completely dissociated. Digestion was terminated with adDMEM/F12, followed by centrifugation, resuspension and filtration through a 100 μm cell strainer. Red blood cell lysis was performed using eBioscience™ RBC Lysis Buffer (Invitrogen, 00-4300-54). The resulting cells were resuspended in a mixture of 70% Matrigel (Corning, 356231) and 30% adDMEM/F12 containing 1% Pen-Strep and 10 μM Y-27632. Every 50 μL dome was plated onto a pre-warmed 24-well plate and incubated upside down in the 37 °C, 5% CO₂ incubator for 30 min to allow the matrigel to solidify. Subsequently, 500 μL organoid culture medium was added to each well. The medium was changed every 2-3 days, and organoids were passaged every 10-14 days depending on their growth.

### Tissue dissociation and 10x single-cell sequencing

For tissues designated for scRNA-seq, enzymatic digestion was performed as described above, followed by filtration through a 40 μm cell strainer and red blood cell lysis. The resulting single-cell suspension was washed and resuspended in 100 μL adDMEM/F12, and cell concentration and viability were assessed using a Luna cell counter. Dead cells were removed based on the proportion of non-viable cells using the MACS Dead Cell Removal Kit (130-090-101), and the remaining cells were recounted. For organoids intended for scRNA-seq, they were dissociated into single cells using TrypLE (Thermo Fisher, 12604013) and subsequently counted. Both types of sequencing samples were resuspended at a concentration of 700-1200 cells/μL after cell counting. The entire mixed cell population was further analyzed without sorting or enrichment for specific cell subtypes. Sequencing and library construction were performed using the 10× Genomics Chromium Next GEM Single Cell 3ʹ Reagent Kits v3.1 according to the manufacturer's protocol. The constructed libraries were subjected to high-throughput sequencing on the Illumina NovaSeq 6000 PE150 platform. All steps were performed according to the manufacturer's standard procedures.

### scRNA-seq data processing, data integration and cluster annotation

The raw data generated from high-throughput sequencing were processed using Cell Ranger (version 8.0.1, 10x Genomics) for quality control and alignment to the reference genome (human: GRCh38). Following the initial quality control by Cell Ranger, further quality filtering was conducted using the Seurat package (version 4.0.0) 17. High-quality cells were retained based on the following criteria: gene numbers > 200, unique molecular identifier (UMI) count > 1000, log_10_GenesPerUMI > 0.7, hemoglobin RNA UMIs < 5%, and less than four times the median mitochondrial UMIs. Additionally, doublet cells were identified and removed using the DoubletFinder package (version 2.0.3) 18. After quality control, the data were normalized using the NormalizeData function in Seurat. Highly variable genes (HVGs) (Top 2000) were identified using the FindVariableGenes function (mean.function = FastExpMean, dispersion.function = FastLogVMR). Batch effects in single-cell expression profiles were corrected using the mutual nearest neighbors (MNN) method in the batchelor package (version 1.6.3) 19. Dimensionality-reduced results were visualized in two-dimensional space using UMAP.

Cell clustering was performed using the FindClusters function at a resolution of 0.8. Cell types were annotated using the SingleR package (version 1.4.1) 20, which calculated the correlation between cell expression profiles to be identified and those in a public reference dataset. Each cell to be identified was assigned the cell type with the highest correlation from the reference dataset. The final cell type was determined by selecting the reference cell type most closely related to the sample's cell expression profile and further manually identified using known cell subtype markers (Table S4). For cell subpopulation clustering, different cell types were extracted individually, and clustering was performed using the respective top 10 principal components (PCs) with different visual inspection resolutions.

### InferCNV estimation

Based on gene expression level from single-cell transcriptomic data, CNV values within each chromosomal region (cutoff 0.1) were estimated by the inferCNV package (version 1.0.4) 21. ECs and fibroblasts were selected as the normal reference cells. Genes were ordered according to their chromosomal positions, and a sliding window of 101 genes was used to calculate the average gene expression values. The expression level of normal cells was used as a control, and the final CNV result file was generated after denoising. Spike-in methods was used to identify the normal epithelial cells and cancer cells 22, 23.

### Gene signatures

Gene list scores were calculated by AddModuleScore function in Seurat. TCGA and CPGEA prostate cancer gene lists were obtained from previous publication by Wang et.al 13. The well-established gene signatures for AR target genes (AR score) and NE, luminal, basal, stem-like and epithelial-mesenchymal transition (EMT) scores were taken from Deng et.al and Dong et.al 14, 24. Cytotoxic genes, including PRF1, GZMB, GZMH, GZMA, NKG7, GNLY, TNFSF10, IFNG and CST7, were obtained from Long et.al 25 (Table S5).

### Pathway analysis

For Gene Set Enrichment Analysis (GSEA) analysis, Hallmark, GO and KEGG term enrichment analyses were performed using GSEA 26 with the H1 Hallmark gene set, C5 GO gene set and C2 KEGG gene set (version 7.2) from the MSigDB database (http://www.gsea-msigdb.org/gsea/msigdb). For Gene Set Variation Analysis (GSVA) analysis, the background gene set file was first downloaded and processed from the KEGG database using the GSEABase package (version 1.44.0). Pathway activity scores for individual cells were then assigned using the GSVA package (version 1.30.0) 27. Finally, the differences in pathway activities between groups were calculated with the LIMMA package (version 3.38.3).

### SCENIC analysis

The SCENIC analysis was performed using the motifs database for RcisTarget and GRNboost (SCENIC version 1.2.4, and AUCell version 1.12.0) with default parameters 28. Each transcription factor (TF) binding motifs were identified based on co-expression. Regulatory activity of each group of regulons was scored by the AUCell package. To evaluate the cell type specificity of each regulon, the regulon specificity score (RSS) 29 based on Jensen-Shannon divergence was calculated using the scFunctions package (https://github.com/FloWuenne/scFunctions/).

### Pseudotime analysis by Monocle

Cell differentiation trajectory inference was performed using the Monocle2 package (version 2.9.0) 30. The importCDS function was used to convert the Seurat object into a CellDataSet object. Genes used for cell ordering (ordering genes, qval < 0.01) were screened by the differentialGeneTest function. Dimensionality reduction and clustering were performed using the reduceDimension function, followed by differentiation trajectory inference using the orderCells function.

### RNA velocity analysis

The spliced and unspliced reads were computed using the Python script velocy.py 31 (https://github.com/velocyto-team/velocy.py) based on the Cell Ranger output folder. RNA velocity values for each gene in each cell were calculated using the R package velocy.R (version 0.6) 31 and projected onto the two-dimensional UMAP space for visualization.

### Cell-cell interaction analysis

Intercellular ligand-receptor interaction analysis was performed using the CellChat R package (version 1.1.3) 32. First, the normalized expression matrix was imported, and a CellChat object was created using the createCellchat function. Preprocessing was conducted with default parameters using identifyOverExpressedGenes, identifyOverExpressedInteractions and projectData functions. Potential ligand-receptor interactions were calculated using computeCommunProb, filterCommunication (min.cells = 10) and computeCommunProbPathway functions. Finally, the intercellular communication network was aggregated using the aggregateNet function.

### Cell cycle analysis

Cell cycle was predicted by the expression of "marker gene pairs" using the Cyclone function of the Scran package (version 1.14.3) 33. The classification was performed using a training dataset and variations in gene expression levels. Cells were classified as being in S phase if both G1 and G2/M phase scores were below 0.5; otherwise, cells were assigned to the cell cycle phase with the higher score.

### Survival analysis

Survival analysis was performed using the online tool GEPIA2.0. RNA-seq and clinical data from TCGA database for prostate adenocarcinoma (PRAD) and other malignancies were used to evaluate the association between genes or gene sets derived from specific cell states and prognosis. To assess the effect of specifically differentially expressed marker genes on tumor progression, tumor samples were divided into two groups with average expression levels of target genes above 75% and below 25%, respectively. Survival curves were plotted using the Kaplan-Meier (KM) method, and statistical significance between the two groups was evaluated using the log-rank test (p-value).

### Hematoxylin-Eosin (H&E) and Immunohistochemistry (IHC) staining

Prostate cancer tissues or matrigel dissolved PDOs were fixed overnight in 10% neutral formalin and embedded in paraffin for sectioning. The sections were stained with H&E (BSBA-4027, ZSGB-BIO) and subjected to IHC staining using antibodies such as PSMA (ab133579, Abcam) and CHGA (TA506095, ZSGB-BIO) following standard protocols (PV-9000, ZSGB-BIO).

### Multiplex immunohistochemistry (mIHC) and IF staining

Double or triple IF staining was performed following the standard staining protocol provided by the TSA kit (AFIHC024, AiFang Biological). The antibodies used for staining included CD4 (#25229, CST), CD8 (#98941, CST), CD56 (#99746, CST), CD31 (ab281583, Abcam), α-SMA (BM0002, Boster), COL1A1 (#72026, CST), ITGA1 (BD-PT5908, Biodragon), TROP2 (ab214488, Abcam) and SOX9 (#82630, CST). The antibodies and corresponding concentrations were detailed in Supplementary Table S6.

### PDOs drug screening

When organoids cultured in a 24-well plate reached a size of 100-150 μm, they were dissolved using TrypLE and digested into single cells. After terminating the digestion, cell density was calculated and adjusted to 400 cells/μL in adDMEM/F12 medium supplemented with 70% Matrigel. A pre-warmed black, clear-bottom 96-well plate (Corning, #3904) was tilted, and 5 μL the cell suspension was pipetted into each well. The plate was inverted and incubated in a 37°C cell incubator for 10 min to allow the matrigel to solidify. Subsequently, 100 μL organoid culture medium (without EGF) was added to each well, and 100 μL PBS was added to the outermost wells of the plate. The growth of organoids was monitored periodically under a light microscope, and the culture medium was replaced every 2-3 days. After 7 days of culture, drug screening was initiated. For each well, 100 μL medium containing drugs or vehicle was added, with 3-5 replicates per group. The organoids were incubated with the drugs for 6 days and the growth was observed regularly using the microscope. On the 7th day post-treatment, organoid viability was assessed using the CellTiter-Glo ® 3D Cell Viability Assay kit (Promega).

### Statistics

All data analyses were performed using GraphPad Prism 10. The significance of differences between groups was determined using the unpaired two-tailed Student's t-test or Mann-Whitney U test. All values were presented as mean ± standard deviation, with statistical significance set at p < 0.05 (*p < 0.05, **p < 0.01, ***p < 0.001, ****p < 0.0001).

## Results

### Significant increase in stromal proportion following hormonal therapy in HRLPC patients

**Figure 1:**
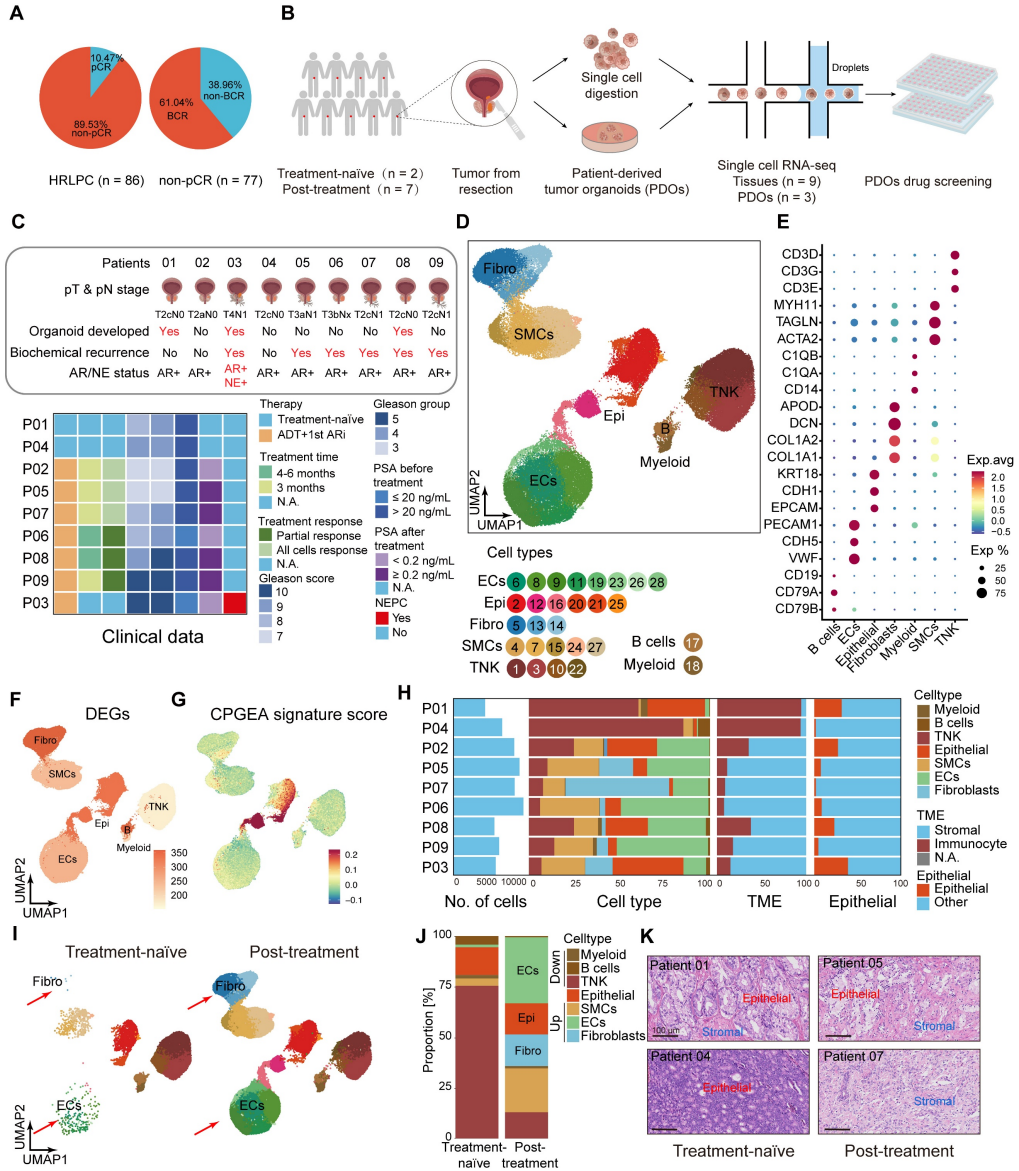
** Hormonal therapy leads to a notable rise in stromal proportion. (A)** Pie charts showing the proportion of pCR and non-pCR patients in the HRLPC cohort after hormonal therapy (left), and the proportion of non-pCR patients who experienced biochemical recurrence (right). **(B)** Summary of experimental methodologies for single-cell analysis of HRLPC. Tissues were isolated from HRLPC patients, and both the tissues and successfully constructed organoids were dissociated into single cells for scRNA-seq. **(C)** Clinical, pathological information and PDOs construction status of the 9 patients. Samples are ordered according to therapy, treatment time, treatment response, Gleason score (GS), Gleason group (GG), PSA before hormonal therapy, PSA after hormonal therapy, NEPC. See also Supplementary Table 1. **(D)** UMAP plot showing identified cell populations from 9 HRLPC patients. **(E)** Dot plot showing representative marker genes across 7 cell subtypes. **(F)** UMAP plot showing the number of DEGs in each cell type. **(G)** UMAP plot showing the CPGEA signature score of all cell types. **(H)** Cell composition distribution for each patient sample. **(I)** UMAP plot showing the distribution of major cell types and treatment groups. **(J)** The stacked bar chart showing the cell composition distribution for each patient sample. **(K)** H&E staining showing the epithelial and stromal proportion change between treatment-naïve and post-treatment groups.

In our constructed HRLPC cohort, the pathologic complete response (pCR) rate of primary tumors in patients receiving hormonal therapy combined with RP was only around 10% (Figure 1A). Importantly, none of the pCR patients experienced biochemical recurrence (BCR) or radiographic recurrence, whereas the BCR rate among non-pCR patients was nearly 60% (Figure 1A). This suggests that the current treatment regimen provides limited efficacy for HRLPC patients. To clarify the changes in the TME of non-pCR patients following hormonal therapy, we performed scRNA-seq analysis on the primary tumor tissues and matched PDOs from nine HRLPC patients (Figure 1B), including two treatment-naïve samples (patient 01, 04), six castration-sensitive prostate cancer (CSPC) samples post-hormonal therapy (patient 02, 05, 06, 07, 08, 09), one amphicrine (AR^+^ and NE^+^) CRPC sample post-hormonal therapy (patient 03) (Figure 1C). Although post-operative prostate-specific antigen (PSA) changes and pathological response evaluations indicated that all patients undergoing hormonal therapy had a treatment response, H&E staining showed that none of patients achieved pCR (Figure 1K; Figure 5H; Figure S1A). After scRNA-seq quality control, we analyzed a total of 91,117 cells across nine samples and identified seven distinct cell types (Figure 1D; Figure S1B-D). Based on established marker genes for major cell types, cells in the merged dataset from nine tissues were annotated as epithelial cells (EPCAM, CDH1, KRT8, KRT18), endothelial cells (ECs) (VWF, CDH5, PECAM1), fibroblasts (APOD, COL1A1, DCN, PDGFRA), smooth muscle cells (MYH11, TAGLN, ACTA2), T cells & NK cells (CD3D, CD3E, NKG7), B cells (CD79A, CD19), and myeloid cells (C1QA, C1QB) (Figure 1D-E, H). We observed that almost all major cell types were present in each primary tumor sample. However, epithelial cells, fibroblasts and immune cells displayed substantial heterogeneity between treatment-naïve and post-treatment samples and were also cell subsets with more differentially expressed genes (DEGs), indicating highly dynamic transcriptional states in these cells after hormonal therapy (Figure 1F). We assessed the cell types using The Cancer Genome Atlas (TCGA) - PRAD and Chinese Prostate Cancer Genome and Epigenome Atlas (CPGEA) signature scores (Figure 1G; Figure S1E). In post-treatment samples, the proportion of immune cells significantly decreased, while the proportion of stromal cells, especially fibroblasts and ECs, significantly increased (Figure 1I-K). The above results suggest that these cell types deserve further in-depth investigation for their roles in shaping the TME of non-pCR cases. Next, we aimed to dissect the transcriptional dynamics of cell subpopulations in the TME post-hormonal therapy.

### Lum stem-like cells become the dominant luminal epithelial subgroup in residual tumors after hormonal therapy

**Figure 2:**
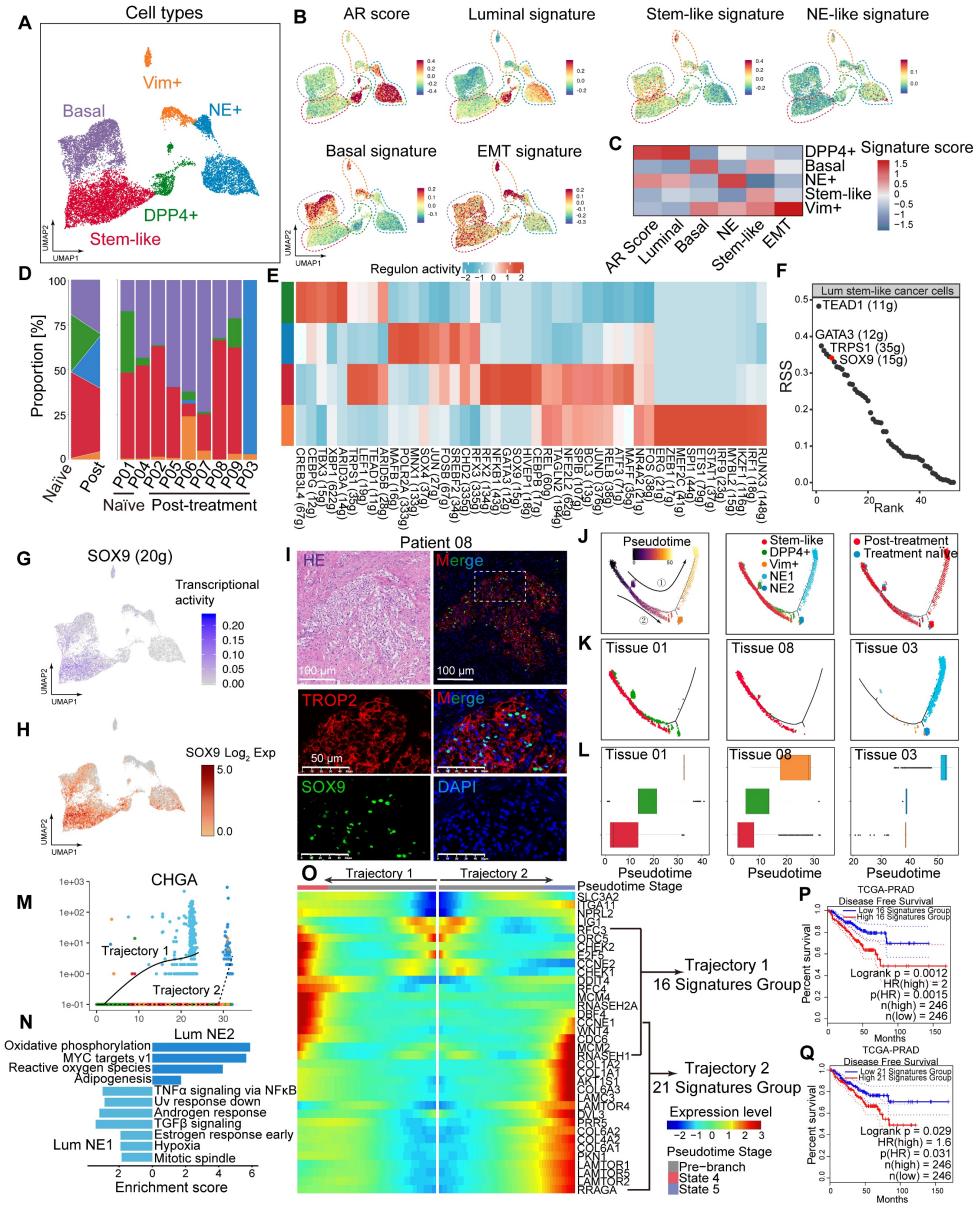
** Lum stem-like cell is the dominant epithelial subgroup in HRLPC after hormonal therapy. (A)** UMAP plot showing the subtypes of epithelial cells from 9 HRLPC patients, including basal cells, Lum DPP4^+^, Lum stem-like, Lum Vim^+^ and Lum NE^+^. **(B)** UMAP plot of single-cell transcriptomic profiles colored by AR, luminal, stem-like, NE-like, basal and EMT gene signature score (z score) for each cell (dot). **(C)** Heat map representing the lineage scores of lineage marker gene signatures in cell subtypes. **(D)** Proportions of each epithelial cell subtypes before and after hormonal therapy and the composition of epithelial cell subtypes for each patient. **(E)** Subpopulation-specific regulons of each epithelial cell subpopulation revealed by SCENIC analysis. **(F)** The regulon specificity score (RSS) ranking plot for Lum stem-like cell subtype. **(G)** The transcriptional activity levels of SOX9 in epithelial cells, cells with the highest transcription level are colored blue. **(H)** The expression level of SOX9 in epithelial cells. Cells with the highest expression level are colored red. **(I)** H&E and mIHC staining showing TROP2^+^ (red) SOX9^+^ (green) and DAPI (blue) in Lum stem-like cells in patient 08. **(J)** Pseudotime trajectory of epithelial cell subtypes by Monocle2. Trajectory is colored by pseudotime (left), cell subtypes (middle), treatment groups (right). **(K-L)** Pseudotime trajectory and boxplot showing the latent time of epithelial cell subtypes by pseudotime. Trajectory is colored by cell subtypes for patient 01, 08 and 03. **(M)** Two-dimensional plots showing the dynamic expression of CHGA along the pseudo-time, colored by epithelial cell subclusters. **(N)** Bar plot for Hallmark pathway enrichment of upregulated genes for Lum NE1 and Lum NE2. **(O)** Heatmap showing the differentially expressed genes across two pseudotime trajectories. **(P-Q)** KM analysis showing the disease-free survival rate of TCGA-PRAD patients with high levels of the two trajectory varied genes using the two-sided log-rank test.

We performed dimensionality reduction on 13,286 epithelial cells identified from the nine primary tumor samples, resulting in 10 subclusters (Figure S2A). Based on reported gene markers for prostate epithelium, we classified these into five epithelial cell subtypes with distinct expression patterns: basal cells and four luminal epithelial subtypes—Lum DPP4^+^, Lum stem-like, Lum Vim^+^ and Lum NE^+^ (Figure 2A; Figure S2B-D). We assessed the epithelial cell subtypes using TCGA - PRAD and CPGEA signature scores (Figure S2E-H). The results showed the lowest scores in basal cells. The signature scores of lineage marker genes for epithelial cells highlighted lineage characteristics of each custom subpopulation (Figure 2B-C; Figure S2I-N). To further identify malignant epithelial cells within these subpopulations, we used ECs and fibroblasts as normal diploid reference and applied inferCNV to predict copy number variations (CNVs) at the single-cell level. Using the spike-in method, benign and malignant epithelial cells were identified in each sample (Figure S3A-C). Benign epithelial cells exhibited lower levels of CNV compared to malignant epithelial cells (Figure S3D). By comparing the composition of epithelial cell clusters before and after hormonal therapy, our results indicated that Lum stem-like and Lum NE^+^ cells emerged as the predominant components among luminal epithelial cell populations in post-treatment samples (Figure 2D). The proportion of Lum DPP4^+^ subtype decreased markedly in post-treatment samples, whereas the Lum stem-like subtype maintained a high proportion before and after treatment (Figure 2D). The shift in luminal epithelial cell subtypes directly reflected the composition of epithelial cells in residual tumor lesions of non-pCR patients following hormonal therapy. The proportion of Lum DPP4^+^ subtype with highest AR score diminished under hormonal treatment pressure, while other luminal epithelial subtypes especially Lum stem-like became predominant, suggesting a need for further analysis of these subtypes. First, we performed SCENIC analysis on the four malignant epithelial cell subtypes (Figure 2E). Regulons associated with stemness and the luminal signature transcription factors (TFs), such as SOX9, GATA3 and NFKB1, showed high regulon specificity score (RSS) in Lum stem-like cells (Figure 2F-G). Meanwhile, we found that SOX9 was relatively highly expressed in the Lum stem-like subgroup (Figure 2H). mIHC staining confirmed the high expression of SOX9 in post-treatment Lum stem-like cells (Figure 2I). SOX9 is known for stemness maintenance, prevention of stem-like cell differentiation in various tumors, and is associated with prostate cancer progression and treatment resistance 34. The high transcriptional activity of these TFs in the above cell clusters validated our definition of residual malignant cell subpopulations associated characterization. To further clarify the trajectory of malignant epithelial changes before and after hormonal therapy, we mapped the pseudotime trajectories of the four luminal epithelial subtypes (Figure 2J). Early state was characterized by Lum stem-like cells and Lum DPP4^+^ cells. This corresponded to the untreated tumors of patients 01 and 04, indicating a pre-treatment phase with an active AR-regulated axis before therapeutic intervention. In trajectory 1, the endpoint consisted predominantly of Lum NE1 NEPC cells, whereas the endpoint of trajectory 2 was composed of Lum stem-like cells, and a small proportion of Lum NE2 cells. These two distinct trajectory endpoints represented the residual epithelial cell states in patients with poor response to hormonal therapy—one type dominated by Lum NE1 cells, representing CRPC patients with characteristic of neuroendocrine small cell carcinoma, and the other dominated by stem-like, EMT cells and a few Lum NE2 cells, representing CSPC patients with a high recurrence rate post-treatment.

We subsequently analyzed all epithelial cells in representative samples from treatment-naïve (patient 01), post-treatment CSPC (patient 08), and post-treatment CRPC (patient 03) cases separately (Figure 2K-L). Patient 01 exhibited Lum DPP4^+^ cells, along with Lum stem-like cells already present. However, the proportion of Lum DPP4^+^ cells in patient 08 significantly decreased, indicating a gradual reduction of Lum DPP4^+^ cells during hormonal therapy. Lum stem-like cells remained abundant as a crucial component of residual tumors in non-pCR patients. In patient 03, Lum NE1 and Lum NE2 cells predominated, representing the final state of treatment resistance (Figure 2K-L). Both trajectory endpoints showed high expression of CHGA (Figure 2M). We further compared NE1 and NE2 cells, finding that NE1 retained relative activation of the AR signaling axis and enrichment in cancer-promoting signaling pathways such as TNF-α and TGF-β, whereas NE2 showed upregulation in oxidative phosphorylation and mitochondrial metabolism (Figure 2N). By aligning the two trajectories with the pseudotime of luminal epithelial cells, we identified the distribution of each cell subtype and noted distinct gene expression changes along trajectories 1 and 2. In trajectory 1, there was a gradual upregulation of genes related to cell cycle and DNA replication, while trajectory 2 was characterized by increased expression of genes related to the mTORC1 pathway (Figure 2O). Genes upregulated at both endpoints were associated with shorter disease-free survival (DFS) in TCGA-PRAD (Figure 2P-Q). Overall, these two trajectories represent distinct malignant outcomes with different molecular characteristics in non-pCR patients post-hormonal therapy. The molecular features of various epithelial cell subtypes provide potential therapeutic targets for further targeted treatments.

### Fibro_CXCL14 and Fibro_SFRP4 are associated with poor prognosis in HRLPC patients

**Figure 3:**
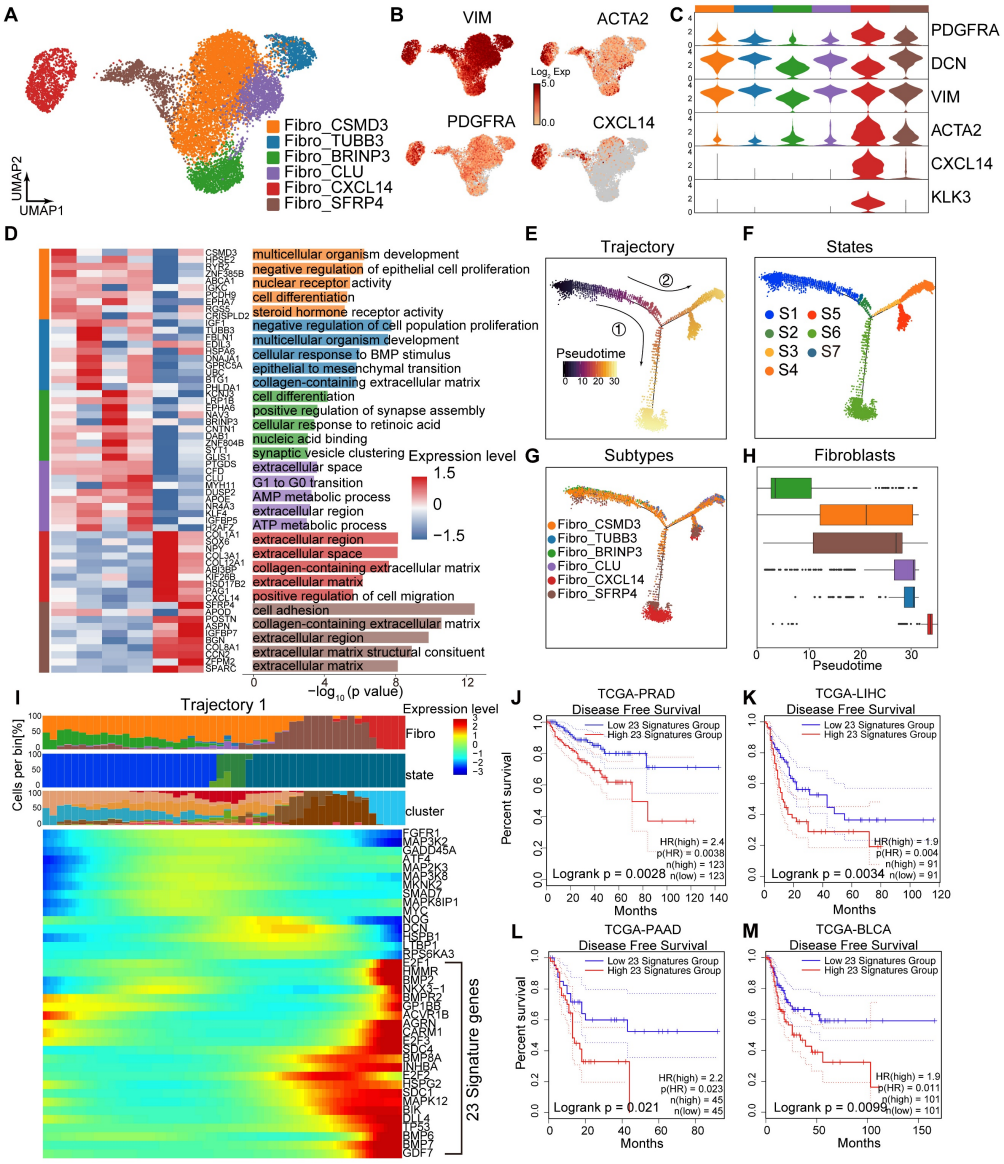
** Fibro_CXCL14 and Fibro_SFRP4 are associated with poor prognosis in HRLPC patients. (A)** UMAP plot showing the subtypes of fibroblasts from 9 HRLPC patients, colored by subtypes. **(B)** The expression level of selected genes. Cells with the highest expression level are colored red. **(C)** Violin plots showing the expression levels of selected markers across fibroblasts subtypes. **(D)** Heatmap (left) displaying the top 10 differentially expressed genes for each defined subtype, and the bar chart (right) showing the GO biological process pathways enriched in each subtype. Significance was determined using a two-sided Wilcoxon rank-sum test. **(E-G)** Pseudotime trajectory of fibroblast subtypes by Monocle2. Trajectory is colored by pseudotime (E), cell states (F) and subtypes (G). **(H)** Boxplot showing the pseudotime of fibroblast subtypes by Monocle2. **(I)** Heatmap showing the differentially expressed genes across pseudotime trajectory 1. The bar charts above represent scaled depictions of subtypes, cell states, and cell clusters along the pseudotime differentiation trajectory. **(J-M)** KM analysis showing the disease-free survival rate of TCGA-PRAD (Prostate adenocarcinoma), LIHC (Liver hepatocellular carcinoma), PAAD (Pancreatic adenocarcinoma) and BLCA (Bladder Urothelial Carcinoma) patients with high and low levels of trajectory 1 fibroblasts 23 genes signature using the two-sided log-rank test.

A total of 11,839 fibroblasts were reclassified into six subclusters: Fibro_CSMD3, Fibro_TUBB3, Fibro_CLU, Fibro_BRINP3, Fibro_CXCL14, and Fibro_SFRP4 (Figure 3A), all of which expressed fibroblasts related genes such as PDGFRA and DCN (Figure 3B-C). The expression patterns, biological functions and proportions of each subcluster varied significantly pre- and post-hormonal therapy, indicating high intratumoral and intertumoral heterogeneity. The Fibro_CXCL14 and Fibro_SFRP4 subclusters were rich in extracellular matrix and cell adhesion functions, with high expression of collagen genes (COL1A1, COL3A1, COL8A1, COL12A1), whereas the Fibro_BRINP3 subcluster was enriched in the cell differentiation pathway (Figure 3D). Using monocle2 for pseudotime analysis, we ordered fibroblasts along a pseudotemporal trajectory, with Fibro_BRINP3 as the starting point, revealing seven distinct states and two trajectory routes (Figure 3E-H). The endpoint of trajectory 1, composed of Fibro_CXCL14 and Fibro_SFRP4 cells, primarily originated from fibroblasts in patients 03 and 06. Genes upregulated along the pseudotime of trajectory 1 were associated with cytokine-cytokine receptor interaction, hormonal therapy resistance and TGF-β signaling pathway (Figure 3I). Survival analysis using 23 highly expressed genes as characteristic fibroblasts signature showed that a high score was associated with poor prognosis in prostate cancer (Figure 3J). This signature was also related to recurrence in other solid tumors include both cancer cells and stromal cell elements (Figure 3K-M).

### Cell-cell communication among Lum stem-like, fibroblasts and ECs is enhanced after hormonal therapy

**Figure 4:**
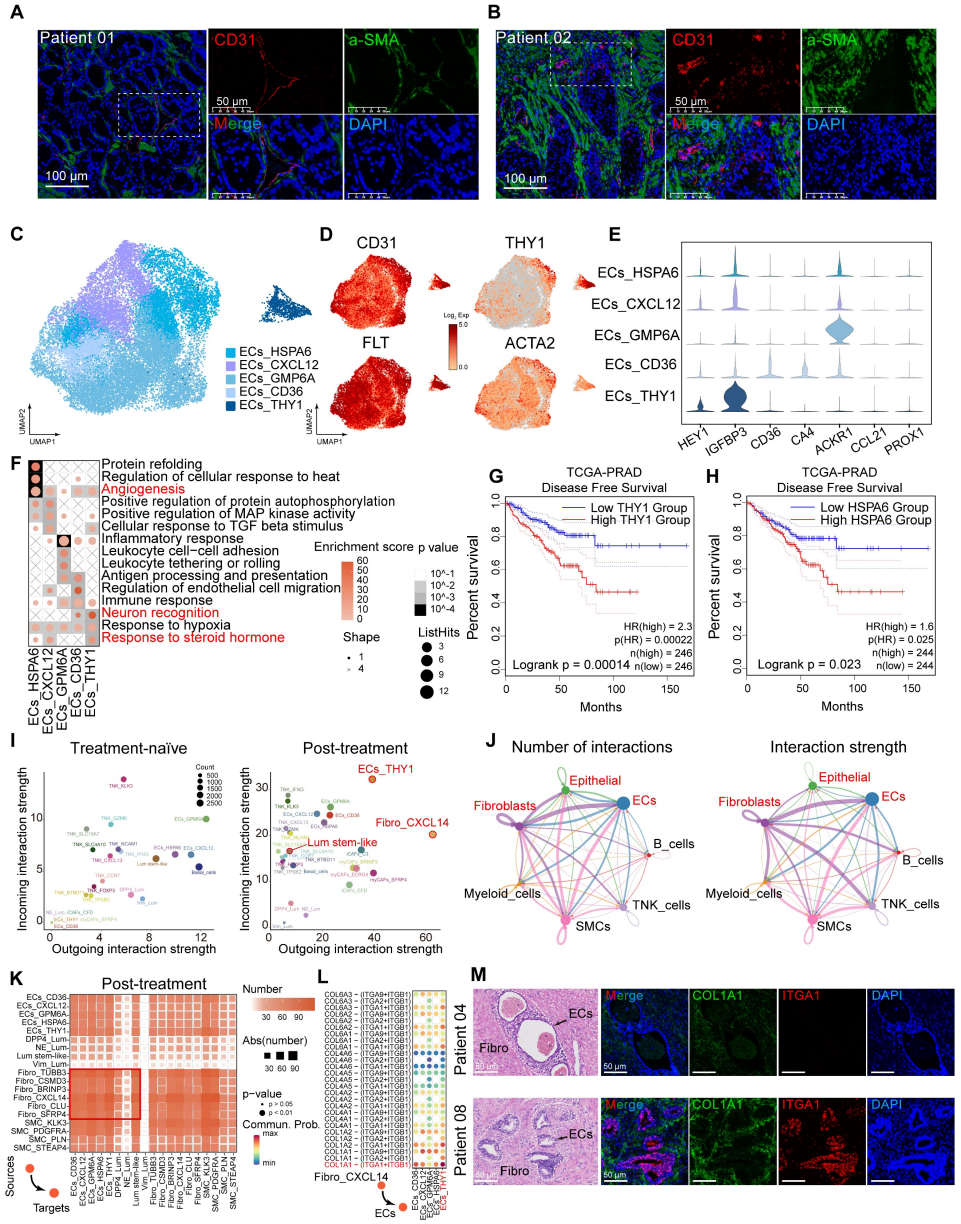
** Cell-cell communication among epithelial cells and the tumor stroma. (A-B)** IF staining showing CD31 (red), a-SMA (green) and DAPI (blue) in treatment-naïve (A) and post-treatment (B) samples. **(C)** UMAP plot showing the 5 subtypes of ECs from 9 HRLPC patients, colored by subtypes. **(D)** The expression level of selected genes. Cells with the highest expression level are colored red. **(E)** Violin plots showing the expression levels of vascular endothelial markers across various EC subtypes. **(F)** Top three most enriched gene ontology (GO) terms for each EC subtype. **(G-H)** KM analysis showing the disease-free survival rate of TCGA-PRAD patients with high and low levels of THY1 (G) or HSPA6 (H) using the two-sided log-rank test. **(I)** The scatter plot showing the incoming and outgoing interaction strengths of each cell type in pre- and post-hormonal therapy samples. **(J)** Interaction number and strength of the 7 annotated cell types after hormonal therapy. **(K)** Heatmap illustrating the cell-cell interaction patterns in post-treatment samples. **(L)** Ligand-receptor pairs between Fibro_CXCL14 and ECs in the collagen pathway, and the communication probability and significance of their communication. **(M)** H&E and IF staining showing COL1A1^+^ (green), ITGA^+^ (red) and DAPI (blue) in treatment-naïve group (patient 04) and post-treatment group (patient 08).

The proportion of stromal cells increased in post-hormonal therapy samples (Figure 1I-J). mIHC staining confirmed an increased proportion of stromal cells, particularly fibroblasts and ECs, within the TME after hormonal therapy (Figure 4A-B; Figure S4A). We analyzed the composition of endothelial cells after treatment, identifying a total of 24,700 ECs divided into five subtypes (Figure 4C; Figure S4B), and observed universal expression of the endothelial marker gene PECAM1 (CD31). High expression of FLT and ACKR1 indicated that most of ECs were of vascular origin (Figure 4D-E). GSVA analysis showed enrichment of pathways related to protein folding, angiogenesis, neuron recognition and steroid response (Figure 4F). Through RNA velocity analysis, we identified unsupervised pseudotime trajectories among various endothelial cell subtypes. ECs_HSPA6 and ECs_THY1 cell subtypes were in the terminal differentiation stage as pseudotemporal mid-to-late stages (Figure S4C-D), and high expression of HSPA6 and THY1 in TCGA-PRAD was associated with poor prognosis (Figure 4G-H). ECs_THY1 cells mainly originated from post-hormonal therapy CRPC patient 03 (Figure 4C; Figure S4B). To investigate cell-cell communication in the TME after hormonal therapy, we used CellChat to compute interaction strengths among cell types before and after therapy and found increased incoming and outgoing signaling strengths for Lum stem-like cells, ECs_THY1 and Fibro_CXCL14 after treatment (Figure 4I). Analysis of the number and strength of ligand-receptor cell communications among seven major cell types revealed that fibroblasts frequently interacted with epithelial cells and ECs after hormonal therapy (Figure 4J). These findings suggest that the reprogrammed interactome landscape of stromal cells post-hormonal therapy may be one reason for failing to achieve pCR in most HRLPC patients. We have demonstrated the simultaneous increase of fibroblasts and ECs and enhanced intercellular communication in the residual TME. Then we further analyzed the ligand-receptor interactions between fibroblasts and other cells within the TME (Figure 4K). Using CellChat to assess the cellular communication among cell subtypes post-hormonal therapy, we found that collagen pathways exhibited the strongest intercellular communication among all ligand-receptor pathways in post-treatment samples (Figure S4E-F). Ligand-receptor interaction analysis suggested potential crosstalk between Fibro_CXCL14 cells and ECs, in which collagen secreted by Fibro_CXCL14 may bind to integrin receptors such as ITGA1 and ITGB1 on ECs (Figure 4L). mIHC staining further confirmed the spatial co-expression of COL1A1 and ITGA1 in post-treatment non-pCR samples (Figure 4M; Figure S4G). In general, the above results indicate that the stromal TME co-constructed by post-treatment fibroblasts, particularly Fibro_CXCL14 cells and ECs_THY1 are associated with hormonal therapy resistance and poor prognosis.

### Decreased immune infiltration and the formation of the immunosuppressive tumor microenvironment post-hormonal therapy

**Figure 5:**
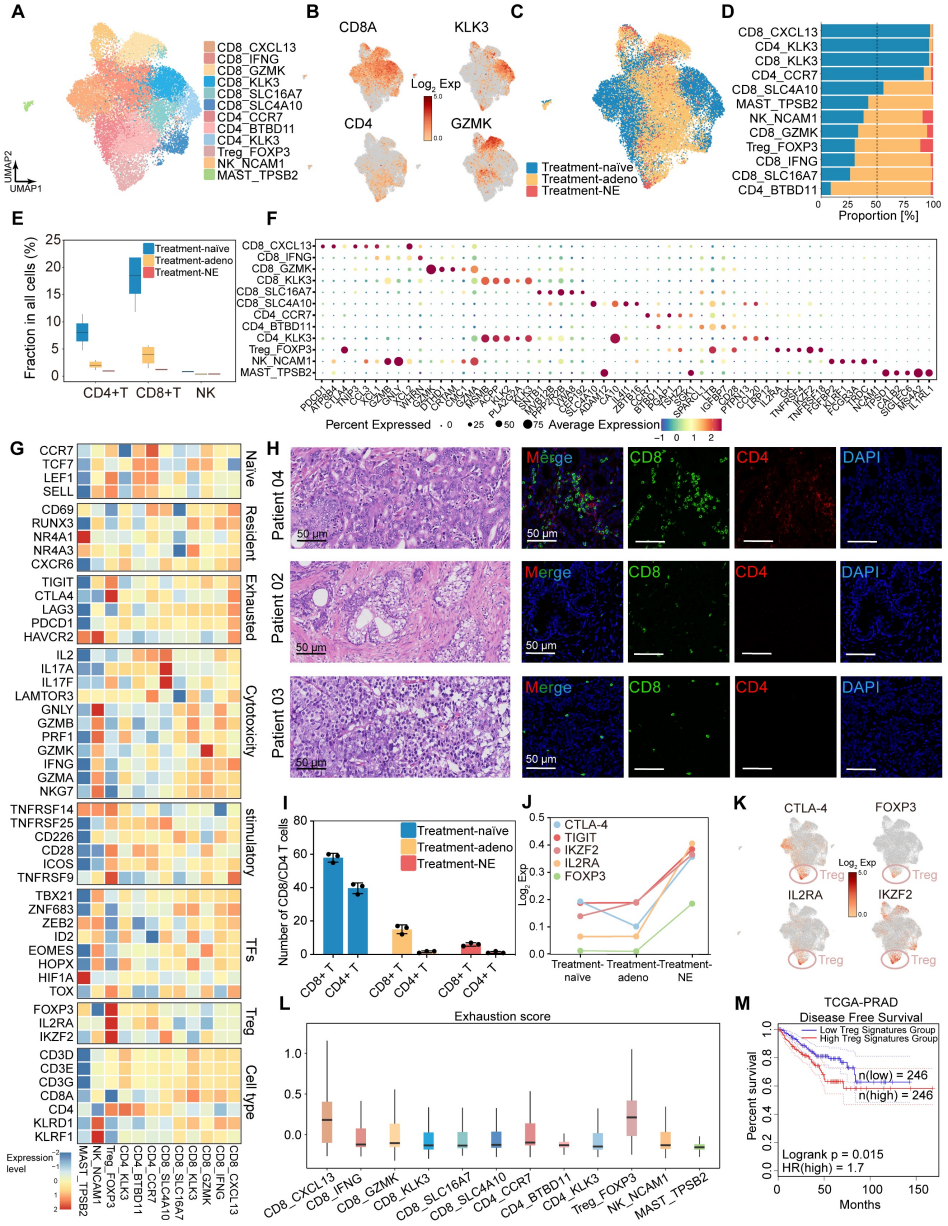
** Hormonal therapy reduces immune infiltration and fosters an immunosuppressive tumor microenvironment. (A)** UMAP plot showing the subtypes of immune cells from 9 HRLPC patients, colored by subtypes. **(B)** The expression level of selected cluster-specific genes. Cells with the highest expression level are colored red. **(C)** UMAP plot showing the treatment groups of immune cells from 9 HRLPC patients, colored by treatment groups. **(D)** The stacked bar chart representing relative abundance of three treatment groups in immune subtypes. **(E)** Boxplot indicating the proportion CD4^+^ T, CD8^+^ T and NK cells in three treatment groups (Treatment-naïve, n = 2; Treatment-adeno, n = 6; Treatment-NE, n = 1). **(F)** Dot plot showing representative marker genes across 12 immune cells subtypes. **(G)** Heatmap indicating the expression of selected gene sets in each immune cell subtype, including naïve, resident, exhausted, cytotoxicity, stimulatory, transcription factors, Treg and cell type. **(H)** H&E and mIHC staining showing CD8^+^ (green), CD4^+^ (red) and DAPI (blue) in treatment-naïve group (patient 04), treatment-adeno group (patient 02) and treatment-NE group (patient 03). **(I)** The bar plots showing the quantification results, n = 3 (9 view fields in total). The error bar indicates standard error of the mean. **(J)** The expression levels (Log_2_ Exp) of T cell exhaustion-related genes and inhibitory immune checkpoint receptor genes across the three treatment groups. **(K)** The expression levels of CTLA-4, FOXP3, IL2RA and IKZF2 in immune cells. Cells with the highest expression level are colored red. **(L)** Box plots showing the exhaustion score of all immune cells subtypes. **(M)** KM analysis showing the disease-free survival rate of TCGA-PRAD patients with high levels of the Treg signature genes using the two-sided log-rank test.

Next, we characterized the transcriptional features of immune cell populations in HRLPC patients before and after hormonal therapy. Unsupervised clustering identified 14 clusters (Figure S4H), which were categorized based on characteristic genes into six CD8^+^ T cell subsets (CD8_CXCL13, CD8_IFNG, CD8_GZMK, CD8_KLK3, CD8_SLC16A7, CD8_SLC4A10), three CD4^+^ T cell subsets (CD4_CCR7, CD4_BTBD11, CD4_KLK3), and subsets of T regulatory (Treg) (Treg_FOXP3), NK (NK_NCAM1), and MAST (MAST_TPSB2) cells (Figure 5A-B). All immune cells were divided into two or three groups based on the treatment phase, with each group of cells present in the samples across different treatment stages (Figure 5C-D; Figure S4I-K). Notably, the proportion of T cells within total cells progressively decreased through the three stages, especially with only a minimal proportion observed in the treatment-NE stage (Figure 5E). Treg_FOXP3 cells were enriched in post-treatment samples, exhibiting co-stimulatory and exhaustible characteristics, with high expression of immunosuppressive factors such as TIGIT and CTLA4 in Treg cells, but lower expression in other clusters (Figure 5F-G). mIHC staining revealed extensive infiltration of CD4^+^ and CD8^+^ T cells in pre-treatment samples, which decreased in post-treatment samples (Figure 5H-I). We detected the average expression of T cell exhaustion-related genes and inhibitory immune checkpoint receptor genes among the three groups, revealing elevated levels in the post-treatment samples, particularly the treatment-NE stage (Figure 5J-K). Exhaustion scores were applied to each subgroup, indicating that Tregs had the highest scores (Figure 5L). Survival analysis using five highly expressed genes as characteristic Treg signature showed that a high score was associated with poor prognosis in prostate cancer (Figure 5M). GSVA analysis of each immune cell subset revealed that CD8^+^ T cells were mainly associated with chemokine receptor binding and NK cell immune regulatory pathways, while Treg cells were enriched in TNFR activity, T cell lineage determination and Toll signaling pathways. Both KLK3^+^ T cells were highly enriched in the Golgi vesicle transport pathway, indicating high vesicular transport activity within the two cell subsets (Figure S4L). NK cells demonstrated significantly upregulated expression of cytotoxic genes, including GNLY, NKG7 and GZMB (Figure S4M-O). Furthermore, scoring using a published signature gene set of cytotoxicity indicated that NK_NCAM1 cells exhibited a relatively high cytotoxicity score, suggesting stronger tumor-killing effect (Figure S4P). Additionally, we identified cell subsets expressing KLK2 and KLK3 in CD4^+^ and CD8^+^ T cells (Figure 5F), a phenomenon proven to be mediated by exogenous KLK3 12, and the subsets significantly decreased post-hormonal therapy (Figure S4K). The above findings suggest that hormonal therapy alters the immune microenvironment of the primary prostate cancer lesion, gradually leading to immune-desert and immune-privileged states.

### PDOs display epithelial subpopulation characteristics that correspond to those observed in tissue samples

**Figure 6:**
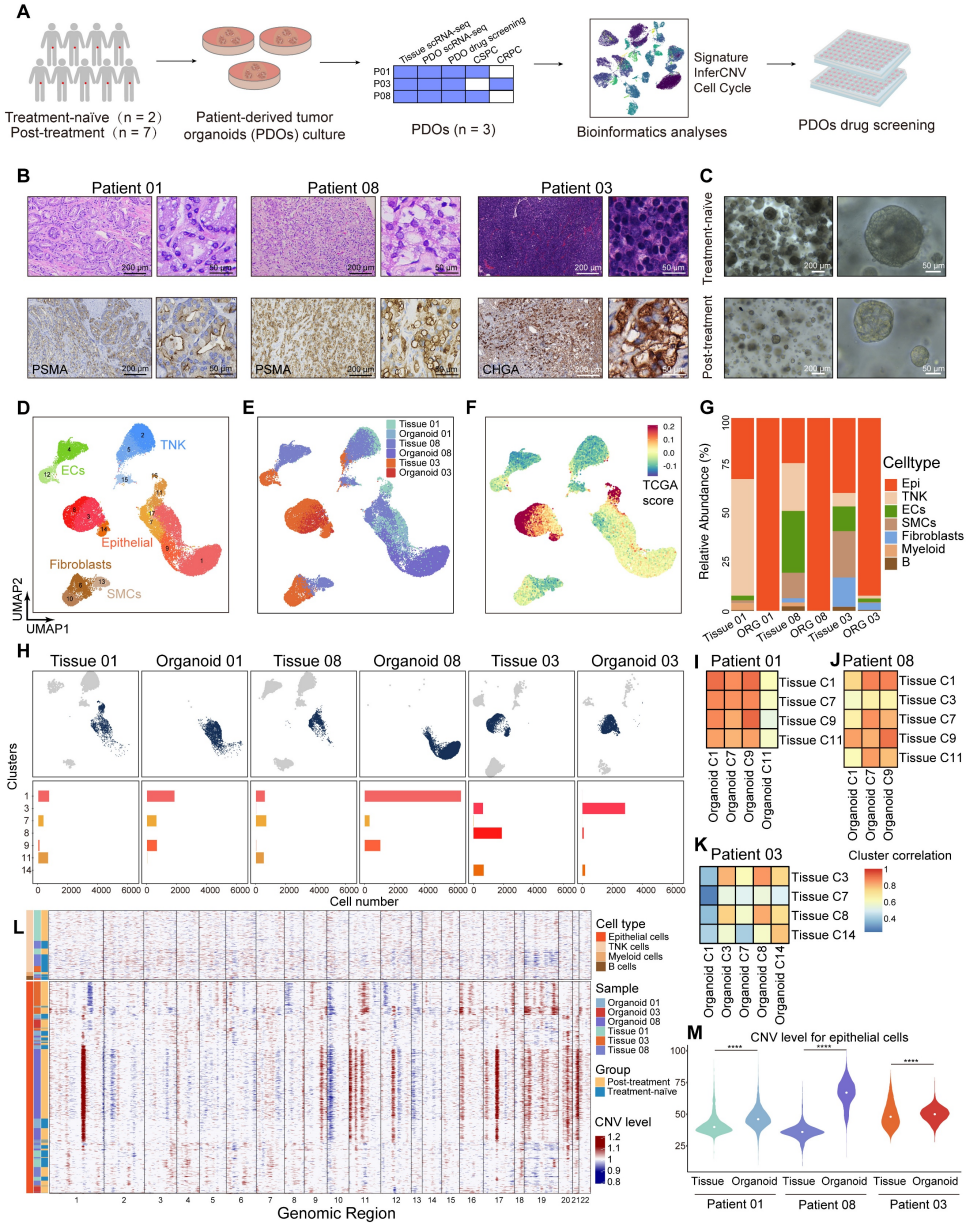
** Single-cell transcriptomic landscape of primary lesions and matched PDOs from HRLPC patients. (A)** Workflow of sample collection, PDOs development, data analysis, and drug screening in this study. **(B)** H&E and IHC staining of tissues from three subgroups: treatment-naïve (patient 01), treatment-adeno (patient 08), and treatment-NE (patient 03). **(C)** Bright-field images of PDOs from treatment-naïve (patient 01) and treatment-adeno (patient 08) groups. **(D-F)** UMAP plots showing the distribution of major cell types, sample names and TCGA signature score of 3 pairs of tissues and PDOs. **(G)** The stacked bar chart representing relative abundance of 6 samples in cell types. **(H)** UMAP plots (top) of 3 pairs of tissues and PDOs. Bar plots (bottom) showing the number of cells in each cluster within each sample. **(I-K)** Heatmap depicting pairwise correlations among epithelial cell clusters derived from 3 pairs of tissues and PDOs. **(L)** inferCNV heatmap with hierarchical clustering from 3 pairs of tissues and PDOs. The top panel indicates a lack of CNV events in reference cells. The bottom panel is the epithelial cells. **(M)** Violin plot of the median and distribution densities of CNV levels in 3 pairs of tissues and PDOs (****p < 0.0001).

Our HRLPC cohort data indicated that the five-year BCR rate approached 60% for HRLPC patients who did not achieve pCR after hormonal therapy. However, existing cell lines poorly model the remaining tumor epithelial cells diversity under hormonal therapy. To elucidate the intratumoral and intertumoral heterogeneity following hormonal therapy in HRLPC patients and explore subsequent treatment strategies, we optimized an established protocol to culture patient-derived prostate cancer organoids from primary lesions obtained from three HRLPC patients (Patient 01, 03, 08) who underwent RP. ScRNA-seq was performed on three organoids (ORG 01, 03, 08) (Figure 6A), representing an untreated CSPC patient at initial diagnosis, a non-pCR CSPC patient post-hormonal therapy, and a non-pCR CRPC patient post-hormonal therapy, respectively (Figure 6B-C). Comprehensive analysis of 36,748 cells from the three pairs of matched primary tumor tissues and organoids via uniform manifold approximation and projection (UMAP) dimensionality reduction revealed 17 subclusters (Figure 6D). The matched tissues and organoids exhibited high distributional concordance, with epithelial cells showing a relatively high TCGA-PRAD signature score (Figure 6E-F). Prostate cancer PDOs were primarily composed of epithelial cells (Figure 6G). Further analysis of the cluster composition of epithelial cell subsets in matched tissue-organoid pairs showed good consistency. Epithelial cells in CSPC tissues and organoids were primarily composed of Clusters 1, 7, 9, and 11, while those in the CRPC tissue and organoid were mainly composed of Clusters 3, 8, and 14 (Figure 6H). The results of high correlation analysis of epithelial cell clusters between tissue and organoid pairs also demonstrated the consistency of the matched samples (Figure 6I-K). Additionally, we conducted inferCNV analysis on immune cells and epithelial cells from the three matched tissue-organoid pairs and determined the CNV landscape (Figure 6L). We found that epithelial cells from organoids showed higher CNV levels than those from tissues (Figure 6M), suggesting competitive survival of different types of malignant epithelium during organoid culture. Overall, organoids derived from residual primary samples of both treatment-naïve and post-hormonal therapy accurately reflect the malignant epithelial characteristics of their parental tissues.

### Molecular characteristics at single-cell resolution suggest that platinum-based chemotherapy is the treatment direction for Lum stem-like dominated patients

**Figure 7:**
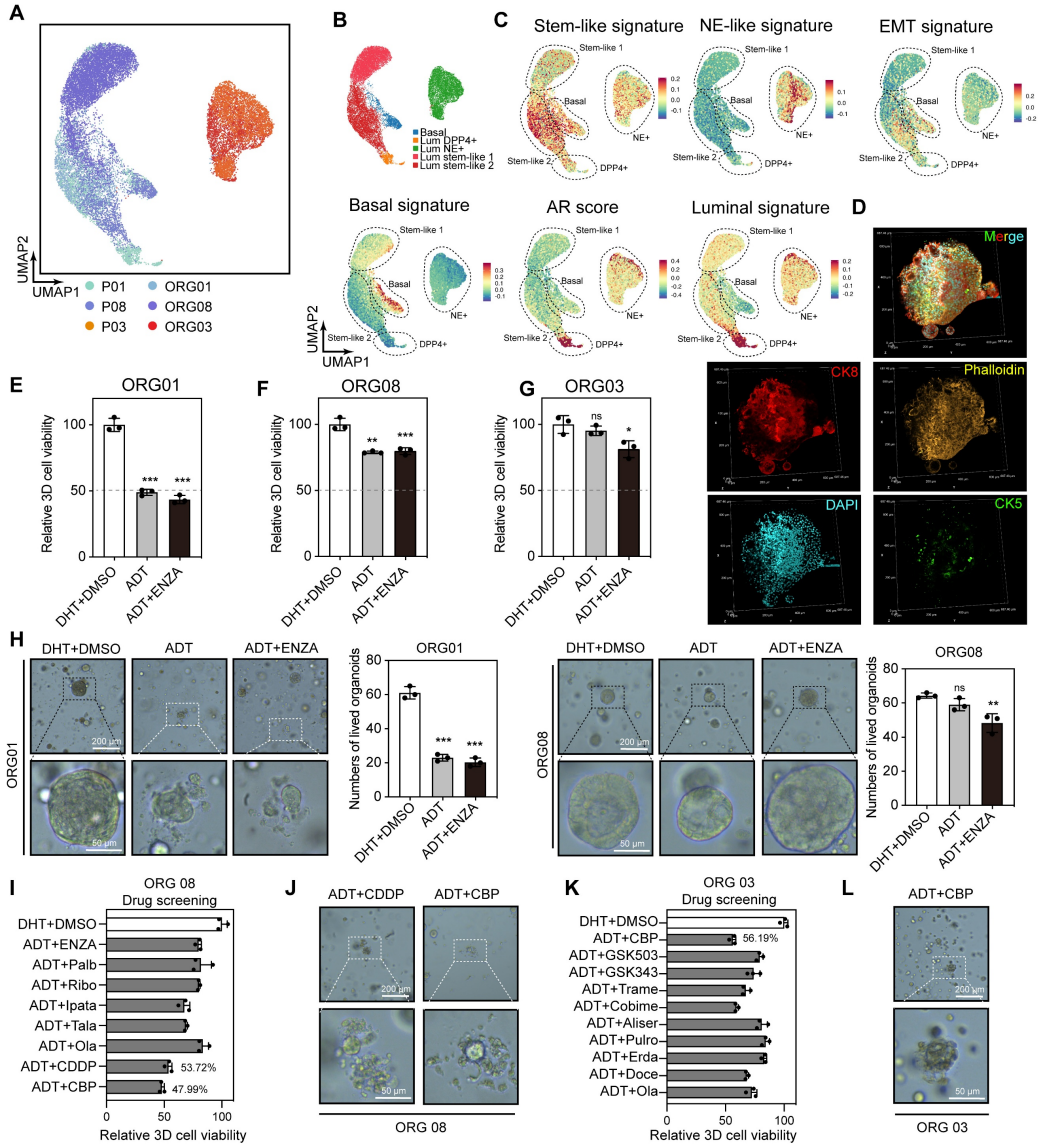
** Single-cell transcriptomic features of epithelial cells in tissues and PDOs, along with organoid drug testing results. (A-B)** UMAP plot showing the subtypes of epithelial cells from 3 pairs of tissues and PDOs, colored by samples and subtypes. **(C)** UMAP plot of single-cell transcriptomic profiles colored by AR, luminal, stem-like, NE-like, basal and EMT gene signature score (z score) for each cell (dot). **(D)** IF staining showing CK5 (green), CK8 (red), Phalloidin (yellow) and DAPI (blue) in treatment-adeno group (patient 08). **(E-G)** Bar plot showing the relative 3D cell viability after treatment with dihydrotestosterone (DHT) + DMSO, ADT, and ADT + ENZA (10 μM) across the three organoid groups: treatment-naïve (patient 01), treatment-adeno (patient 08), and treatment-NE (patient 03). Bars represent mean ± s.e.m. for at least three independent experiments. * P < 0.05, ** P < 0.01, *** P < 0.001. **(H)** PDOs after treatment with DHT + DMSO, ADT, and ADT + ENZA (10 μM). **(I-L)** Bar plot and organoids bright-field images showing the relative 3D cell viability after treatments in treatment-adeno (I-J, patient 08), and treatment-NE (K-L, patient 03).

Our previous analysis of primary tumor epithelial cells indicated that the main components of residual tumors following hormonal therapy were Lum stem-like and Lum NE^+^ cells, both of which showed molecular characteristics associated with treatment resistance and recurrence in prostate cancer. To explore further treatment options for non-pCR patients, we performed dimensionality reduction on 21,095 epithelial cells from three matched tissue and organoid pairs (Figure 7A; Figure S5A-C). Based on basal, luminal, stemness and NE signature scores, we redefined these cells into five subtypes (Basal, Lum DPP4^+^, Lum stem-like 1, Lum stem-like 2, Lum NE^+^) (Figure 7B-C). Immunofluorescence (IF) staining of organoids showed that they primarily consisted of luminal epithelial cells expressing CK8, with absent CK5 expression (Figure 7D). Consistent with findings from the nine tissue samples, a population of Lum stem-like cells was present in both pre- and post-treatment CSPC samples, and continued to persist in residual lesions despite the decrease in Lum DPP4^+^ cells under hormonal therapy pressure (Figure 7A-B). We found that ADT combined with enzalutamide (ENZA) (10 μM) still showed good efficacy for initially untreated CSPC PDOs (Figure 7E), whereas neither non-pCR CSPC nor CRPC PDOs post-treatment achieved half-maximal inhibition, indicating a shift in dominant subclones of epithelial cells in post-treatment samples. Specifically, Lum stem-like and Lum NE^+^ cells exhibited resistance to ADT and ENZA (Figure 7F-H). Cell cycle analysis of three organoid samples revealed a high proportion of S/G2M phase cells in ORG 01 and ORG 03, while ORG 08 composed mainly of Lum stem-like 1 and Lum stem-like 2 cells, also retained a high proportion of S phase cells (Figure S5D-G). These results indicated that most cells in PDOs from both treatment-naïve and post-treatment patients were in a dividing phase, and a portion of cells in post-treatment CSPC patients also remained in this period. We conducted drug screening on ORG 03 and ORG 08, using hormonal therapy in combination with other treatment regimens such as chemotherapy and several classes of drugs commonly used in phase II-III clinical trials. We found that ORG 01 and ORG 08 were more sensitive to cisplatin and carboplatin, and ORG 03 showed sensitivity to carboplatin (a first-line medication for small cell lung cancer) (Figure 7I-L; Figure S5H-I). The combined analysis of single-cell transcriptomics data and organoid drug screening results indicated that residual tumors in non-pCR patients after hormonal treatment had been shaped by treatment pressure. Many potentially resistant cells, like Lum stem-like and Lum NE^+^ cells, survived and exhibited significant heterogeneity, which limited the effectiveness of ADT combined with AR signaling inhibitors. Platinum-based chemotherapy like cisplatin and carboplatin may be effective for patients predominantly composed of Lum stem-like cells and Lum NE^+^ cells dominant tumors.

## Discussion

HRLPC patients have a high prevalence and poor prognosis in China 35. Hormonal therapy as the cornerstone has been recommended as the treatment regimen for HRLPC patients, while approximately 50% patients experience recurrence within five years 36. For non-pCR patients after hormonal therapy, exploring the changes in malignant epithelial cells and the TME post-treatment could provide key insights for further treatment strategies. Currently, numerous studies on localized prostate cancer and CRPC have widely utilized bulk transcriptomics, scRNA-seq and proteomics, and these multi-omics data results provide significant insights into the molecular characteristics of prostate cancer at these stages 37-39. However, for HRLPC patients, there is a scarcity of single-cell resolution studies focused on this specific sample type, especially regarding intra- and inter-tumoral heterogeneity in residual tumors and changes in the TME after hormonal therapy. In this study, we aimed to use radical resection samples from HRLPC patients pre- and post-hormonal therapy to provide a comprehensive single-cell transcriptomic landscape, characterizing the epithelial cells and TME in non-pCR patients. This study details the cellular diversity and heterogeneity of tumor, stromal and immune components in post-treatment HRLPC tissues, as well as in matched PDOs. The results reveal the molecular characteristics of various cell subpopulations at single-cell resolution that contribute to the high recurrence rate in non-pCR patients.

ScRNA-seq results of transgenic mice prostate cancer offer certain reference effect on human prostate cancer. Recent scRNA-seq studies in mice have shown that normal prostate luminal epithelial cells exhibit a degree of adaptability after castration surgery, and a stem cell-like luminal subpopulation has been identified 40, which has also been observed in human benign prostatic hyperplasia tissues 41. Our study identified a population of Lum stem-like cells in HRLPC patients, which became the dominant subpopulation in non-pCR patients after treatment. Unlike the extremely rare CRPC-like cells that existed in pre-treatment samples proposed by Cheng et al. 42, Lum stem-like cells expressed stemness transcription regulators with high transcriptional activity, such as SOX9 and stem cell-like genes (e.g., Ly6d and Tacstd2/Trop2), which were closer to the Luminal-C cells in mouse models 43. Our data suggested that this cell population responded poorly to hormonal therapy, exhibited low AR and NE pathway scores, and was predominantly in the early and middle stages of the pseudotemporal trajectory, prior to the NEPC phase, which may be one of the main reasons for the high recurrence rate in non-pCR HRLPC patients after hormonal therapy. Additionally, we identified a population of Vim^+^ epithelial cells that increased in proportion after hormonal therapy. Pseudotime analysis suggested that these cells emerged in the middle and late stages of treatment and gradually lost epithelial markers. This subpopulation displayed high transcriptional activity of EMT-related regulons such as ETS1, MEF2C and KLF2 44. ETS1, a member of the ETS TFs family, has been shown to promote the acquisition of invasiveness, EMT, angiogenesis and drug resistance in cancer cells. In prostate cancer, ETS1 can promote EMT processes through TGF-β signaling pathway in cancer cells, involving in cancer progression 45, 46. NEPC, as the terminal state of CRPC, represents a subset of patients who are completely resistant to hormonal therapy. In our study, Lum NE^+^ cells were predominantly present in the patient 03, and a portion of Lum NE^+^ cells was identified in the non-pCR CSPC patient post-treatment (patient 06), indicating treatment-induced NEPC (tNEPC) differentiation 47, 48. In the epithelial cell pseudotime trajectory, both Lum NE1 and Lum NE2 cells appeared at the pseudotime terminal stage of luminal epithelial cells yet displayed distinct molecular characteristics. Lum NE1 cells exhibited dual-positive (AR^+^ and NE^+^) cells with sustained activation of the AR signaling axis, while Lum NE2 cells demonstrated features of metabolic reprogramming, with upregulation of genes related to oxidative phosphorylation. Our findings suggest that tNEPC exhibit significantly intra- and inter-tumoral heterogeneity, which may be a critical factor in the limited survival benefit of current therapies for NEPC patients.

Fibroblasts and ECs, as crucial components of the TME, have been shown to play a significant role in prostate cancer recurrence, metastasis and therapeutic resistance 49-52. In our samples, these two cell types were markedly increased in samples following hormonal therapy, and a significant increase in stromal components post-treatment was also confirmed by H&E and mIHC staining of matched tissue samples. The high recurrence rate in non-pCR patients drew our attention to the interactions between stromal components and their impact on residual tumor cells. High expression of THY1 in ECs is associated with poor prognosis across various cancers 53, and the increase in vascular ECs post-treatment likely provides essential blood supply to residual tumors, which may contribute to prostate cancer recurrence following hormonal therapy. Fibroblasts have become central members of the TME, acting as fertile "soil" that supports tumor cell "seeds" in multiple ways 54. Our findings indicated a significant increase in fibroblasts promoting tissue remodeling post-hormonal therapy. Two subtypes, Fibro_CXCL14 and Fibro_SFRP4 at the end of pseudotime trajectory 1, with significant enrichment in pathways related to extracellular matrix remodeling, cell migration and adhesion were defined. These findings suggested that fibroblasts underwent reprogramming after hormonal therapy, creating a stromal environment more conducive to tumor survival. The fibroblasts gene set we constructed achieved a good predictive value for recurrence in prostate cancer and other solid tumors. Intercellular communication analysis revealed that fibroblasts exhibited extensive signaling interactions with epithelial cells and other stromal components. Fibroblasts highly expressed type I collagen genes such as COL1A1 and COL1A2, and secreted collagen into the TME after hormonal therapy. These proteins have been demonstrated in previous studies to be associated with tumor progression and reduced immune infiltration in various cancers 55, 56. For example, the network structure formed by collagen and integrins facilitates the migration of stromal cells towards the glands and encapsulates the glands 57. The accumulation of fibroblasts and ECs in residual tumor lesions post-hormonal therapy forms a barrier, reducing immune cell infiltration around glands and contributing to an hormonal therapy-induced immune-desert state in prostate cancer 58.

Numerous phase II and III clinical trials have attempted immunotherapy for metastatic CRPC patients, but the efficacy has been generally disappointing 59-61. Single-cell transcriptomic studies of the primary sites of prostate cancer have indicated that the TME is relatively immune-depleted, which may explain the poor efficacy of immunotherapy in prostate cancer 12, 40, 62. This study revealed the changes in the immune microenvironment of the primary prostate cancer lesion in HRLPC patients after hormonal therapy. In the pre-treatment samples, immune cell proportions were significantly higher than previously reported sparse immune infiltration in primary prostate cancer lesions 12, 63. However, we found that the proportion of immune cells, particularly T cells, reduced significantly in seven HRLPC patients post-hormonal therapy compared with pre-treatment patients, with some non-pCR cases (e.g., patient 03, 06) showing an “immune-desert” phenotype. This contrasted with previous findings of substantial T-cell infiltration in the TME following ADT treatment 64, 65. Additionally, similar to previous studies, a population of KLK3-expressing CD4^+^ and CD8^+^ T cells was also identified in our study 12. These cells exhibited high levels of Golgi vesicle transport and were associated with immune responses mediated by myeloid cells and mast cells, while the proportion of these cells significantly decreased post-hormonal therapy, potentially due to the suppression of the AR signaling pathway. On the other hand, cell populations that increased post-treatment included immunosuppressive Treg_FOXP3 cells and senescent CD8_GZMK cells, which highly expressed immunosuppressive related molecules such as TIGIT, CTLA-4 and LAG3. This suggests that reduced immune infiltration and the emergence of immune-privileged state after hormonal therapy may contribute to the failure of immunotherapy. Previous studies have shown that there is also a high level of immune infiltration in metastases of treatment-naïve metastatic CSPC patients, and T-cell infiltration could be induced and maintained in these lesions when treated with ADT combined with immunotherapy 66. Collectively, these findings suggest that there is a subset of prostate cancer patients with initially high immune infiltration, while who transition to an immune-desert or immunosuppressive state after hormonal therapy. Identifying these patients at initial treatment could help determine those who may benefit from combined immunotherapy strategies.

The application of organoid construction in prostate cancer has become increasingly matured, and breakthroughs have been made in lineage plasticity research using genetically engineered mouse-derived prostate organoids 43, 67. Since Gao et al. successfully established PDOs from metastatic prostate cancer, the application of prostate cancer PDOs has gradually attracted considerable attention 15, 68, 69. However, there remains a lack of in-depth single-cell analysis of PDOs. To our knowledge, the only study performing scRNA-seq on primary prostate cancer organoids contained only a small number of tumor cells, with the majority composed of normal epithelial cells and exhibiting low concordance with the paired parental tissues 62. In our study, we successfully constructed organoids from primary lesions of prostate cancer patients before and after hormonal therapy and conducted scRNA-seq on early-passage organoids and matched tissues. Our results provided a comprehensive single-cell transcriptomic profile of PDOs and matched tissues, demonstrating a high degree of concordance. The cell subpopulations within PDOs corresponded well to those in the parental tissues, effectively reflecting the molecular characteristics of epithelial cells within the original tissues. For non-pCR patients with high interpatient heterogeneity following hormonal therapy, the construction of homologous PDOs provides valuable guidance for formulating subsequent treatment regimens 70, 71. By integrating single-cell transcriptomic analysis with drug screening on organoids, we identified potential reasons for the persistence of residual tumor in non-pCR patients after hormonal therapy. Additionally, alternative treatment strategies such as CDK4/6 inhibitors, PARP inhibitors, BET inhibitors and platinum-based chemotherapy were tested in PDOs. CDK4/6 inhibitors, such as Palbociclib, are currently being investigated in clinical trials for mCRPC and RB-positive metastatic prostate cancer. Although their combined efficacy with ADT was suboptimal in organoids in this study, CDK4/6 inhibitors have been shown to enhance tumor immune response and increase tumor-infiltrating lymphocytes. This suggests a potential synergistic effect when combined with immune checkpoint inhibitors in the immunosuppressive microenvironment formed after ADT treatment in prostate cancer 72. In addition, we found that platinum-based chemotherapy exhibited significant inhibitory and cytotoxic effects on organoids predominantly composed of Lum stem-like cells and Lum NE^+^ cells. Currently, platinum plus etoposide combination therapy is recommended in clinical practice for neuroendocrine-differentiated or small cell prostate cancer; however, no phase 3 clinical trial data are available to support these recommendations 73. Existing studies suggest that platinum-based therapies have shown promising preliminary results in mCRPC 74. Further investigation into the efficacy of platinum-based therapies in specific CSPC and CRPC subtypes is crucial for optimizing their clinical application. The results may offer guidance for future clinical decisions in HRLPC patients and potentially reduce recurrence rates.

Furthermore, we found that current prostate organoid culture conditions predominantly supported epithelial cell preservation from parental tissues after several passages, yet struggled to retain the original TME, such as fibroblasts and immune cells. Currently, co-culture with PDOs requires the addition of exogenous cells 51, 75, adding complexity to the culture process. Therefore, exploring primary organoid construction and culture paradigms that can directly simulate interactions between the TME and organoids from primary lesions is of great significance for in-depth analysis of the relationship between stroma and tumor cells 76.

Since biopsy samples were primarily used for pathological diagnosis, paired longitudinal scRNA-seq analysis of samples before and after hormonal therapy was not conducted. Additionally, given the interpatient heterogeneity in prostate cancer, the data from seven post-treatment non-pCR patients may not fully represent the entire non-pCR patient population. However, this remains the largest scRNA-seq dataset of non-pCR samples after hormonal therapy from HRLPC patients to date.

## Conclusions

In summary, our study provides a unique single-cell transcriptomic dataset of primary tumor lesions and matched organoids from non-pCR HRLPC patients after hormonal therapy, which encompasses diverse cellular components within the TME and has been validated at the organoid level. This data facilitates a deeper understanding of recurrence mechanisms in non-pCR patients following hormonal therapy and offers valuable resources to guide subsequent precision treatment.

## Supplementary Material

Supplementary figures.

Supplementary tables.

## Abbreviations

ADT: androgen deprivation therapy
AMPC: amphicrine prostate cancer
AR: androgen receptor
ARi: androgen receptor inhibitors
BCR: biochemical recurrence
CNVs: copy number variations
CPGEA: Chinese Prostate Cancer Genome and Epigenome Atlas
CRPC: castration-resistant prostate cancer
CSPC: castration-sensitive prostate cancer
DEGs: differentially expressed genes
DFS: disease-free survival
DHT: dihydrotestosterone
ECs: endothelial cells
EMT: epithelial-mesenchymal transition
ENZA: enzalutamide
GO: gene ontology
GSEA: Gene Set Enrichment Analysis
GSVA: Gene Set Variation Analysis
H&E: Hematoxylin and Eosin
HRLPC: high-risk/locally advanced prostate cancer
HVGs: highly variable genes
IF: immunofluorescence
KM: Kaplan-Meier
mIHC: multiplex immunohistochemistry
MNN: mutual nearest neighbors
NEPC: neuroendocrine prostate cancer
pCR: pathological complete response
PCs: principal components
PDOs: patient-derived organoids
Pen-Strep: penicillin-streptomycin
PRAD: prostate adenocarcinoma
RP: radical prostatectomy
RSS: regulon specificity score
RT: radical radiotherapy
scRNA-seq: single-cell RNA sequencing
TCGA: The Cancer Genome Atlas
TFs: transcription factors
TME: tumor microenvironment
tNEPC: treatment-induced NEPC
Treg: T regulatory
UMAP: uniform manifold approximation and projection
UMI: unique molecular identifier

## References

[1] Helgstrand JT, Roder MA, Klemann N, Toft BG, Brasso K, Vainer B (2017). Diagnostic characteristics of lethal prostate cancer. Eur J Cancer.

[2] Mateo J, Seed G, Bertan C, Rescigno P, Dolling D, Figueiredo I (2020). Genomics of lethal prostate cancer at diagnosis and castration resistance. J Clin Invest.

[3] Hamdy FC, Donovan JL, Lane JA, Metcalfe C, Davis M, Turner EL (2023). Fifteen-Year Outcomes after Monitoring, Surgery, or Radiotherapy for Prostate Cancer. N Engl J Med.

[4] Mottet N, van den Bergh RCN, Briers E, Van den Broeck T, Cumberbatch MG, De Santis M (2021). EAU-EANM-ESTRO-ESUR-SIOG Guidelines on Prostate Cancer-2020 Update. Part 1: Screening, Diagnosis, and Local Treatment with Curative Intent. Eur Urol.

[5] Zhu Y, Mo M, Wei Y, Wu J, Pan J, Freedland SJ (2021). Epidemiology and genomics of prostate cancer in Asian men. Nat Rev Urol.

[6] Chen R, Ren S, Chinese Prostate Cancer C, Yiu MK, Fai NC, Cheng WS (2014). Prostate cancer in Asia: A collaborative report. Asian J Urol.

[7] Dee EC, Nezolosky MD, Chipidza FE, Arega MA, Butler SS, Sha ST (2020). Prostate cancer-specific mortality burden by risk group among men with localized disease: Implications for research and clinical trial priorities. Prostate.

[8] Wala J, Nguyen P, Pomerantz M (2023). Early Treatment Intensification in Metastatic Hormone-Sensitive Prostate Cancer. J Clin Oncol.

[9] Tilki D, van den Bergh RCN, Briers E, Van den Broeck T, Brunckhorst O, Darraugh J (2024). EAU-EANM-ESTRO-ESUR-ISUP-SIOG Guidelines on Prostate Cancer. Part II-2024 Update: Treatment of Relapsing and Metastatic Prostate Cancer. Eur Urol.

[10] Sun R, A J, Yu H, Wang Y, He M, Tan L (2024). Proteomic landscape profiling of primary prostate cancer reveals a 16-protein panel for prognosis prediction. Cell Rep Med.

[11] Dong B, Xu JY, Huang Y, Guo J, Dong Q, Wang Y (2024). Integrative proteogenomic profiling of high-risk prostate cancer samples from Chinese patients indicates metabolic vulnerabilities and diagnostic biomarkers. Nat Cancer.

[12] Chen S, Zhu G, Yang Y, Wang F, Xiao YT, Zhang N (2021). Single-cell analysis reveals transcriptomic remodellings in distinct cell types that contribute to human prostate cancer progression. Nat Cell Biol.

[13] Wang Z, Wang T, Hong D, Dong B, Wang Y, Huang H (2022). Single-cell transcriptional regulation and genetic evolution of neuroendocrine prostate cancer. iScience.

[14] Dong B, Miao J, Wang Y, Luo W, Ji Z, Lai H (2020). Single-cell analysis supports a luminal-neuroendocrine transdifferentiation in human prostate cancer. Commun Biol.

[15] Gao D, Vela I, Sboner A, Iaquinta PJ, Karthaus WR, Gopalan A (2014). Organoid cultures derived from patients with advanced prostate cancer. Cell.

[16] Drost J, Karthaus WR, Gao D, Driehuis E, Sawyers CL, Chen Y (2016). Organoid culture systems for prostate epithelial and cancer tissue. Nat Protoc.

[17] Hao Y, Hao S, Andersen-Nissen E, Mauck WM 3rd, Zheng S, Butler A (2021). Integrated analysis of multimodal single-cell data. Cell.

[18] McGinnis CS, Murrow LM, Gartner ZJ (2019). DoubletFinder: Doublet Detection in Single-Cell RNA Sequencing Data Using Artificial Nearest Neighbors. Cell Syst.

[19] Haghverdi L, Lun ATL, Morgan MD, Marioni JC (2018). Batch effects in single-cell RNA-sequencing data are corrected by matching mutual nearest neighbors. Nat Biotechnol.

[20] Aran D, Looney AP, Liu L, Wu E, Fong V, Hsu A (2019). Reference-based analysis of lung single-cell sequencing reveals a transitional profibrotic macrophage. Nat Immunol.

[21] Puram SV, Tirosh I, Parikh AS, Patel AP, Yizhak K, Gillespie S (2017). Single-Cell Transcriptomic Analysis of Primary and Metastatic Tumor Ecosystems in Head and Neck Cancer. Cell.

[22] Ge G, Han Y, Zhang J, Li X, Liu X, Gong Y (2022). Single-Cell RNA-seq Reveals a Developmental Hierarchy Super-Imposed Over Subclonal Evolution in the Cellular Ecosystem of Prostate Cancer. Adv Sci (Weinh).

[23] Maynard A, McCoach CE, Rotow JK, Harris L, Haderk F, Kerr DL (2020). Therapy-Induced Evolution of Human Lung Cancer Revealed by Single-Cell RNA Sequencing. Cell.

[24] Deng S, Wang C, Wang Y, Xu Y, Li X, Johnson NA (2022). Ectopic JAK-STAT activation enables the transition to a stem-like and multilineage state conferring AR-targeted therapy resistance. Nat Cancer.

[25] Long Z, Sun C, Tang M, Wang Y, Ma J, Yu J (2022). Single-cell multiomics analysis reveals regulatory programs in clear cell renal cell carcinoma. Cell Discov.

[26] Subramanian A, Tamayo P, Mootha VK, Mukherjee S, Ebert BL, Gillette MA (2005). Gene set enrichment analysis: a knowledge-based approach for interpreting genome-wide expression profiles. Proc Natl Acad Sci U S A.

[27] Hanzelmann S, Castelo R, Guinney J (2013). GSVA: gene set variation analysis for microarray and RNA-seq data. BMC Bioinformatics.

[28] Aibar S, Gonzalez-Blas CB, Moerman T, Huynh-Thu VA, Imrichova H, Hulselmans G (2017). SCENIC: single-cell regulatory network inference and clustering. Nat Methods.

[29] Suo S, Zhu Q, Saadatpour A, Fei L, Guo G, Yuan GC (2018). Revealing the Critical Regulators of Cell Identity in the Mouse Cell Atlas. Cell Rep.

[30] Trapnell C, Cacchiarelli D, Grimsby J, Pokharel P, Li S, Morse M (2014). The dynamics and regulators of cell fate decisions are revealed by pseudotemporal ordering of single cells. Nat Biotechnol.

[31] La Manno G, Soldatov R, Zeisel A, Braun E, Hochgerner H, Petukhov V (2018). RNA velocity of single cells. Nature.

[32] Jin S, Guerrero-Juarez CF, Zhang L, Chang I, Ramos R, Kuan CH (2021). Inference and analysis of cell-cell communication using CellChat. Nat Commun.

[33] Lun AT, McCarthy DJ, Marioni JC (2016). A step-by-step workflow for low-level analysis of single-cell RNA-seq data with Bioconductor. F1000Res.

[34] Nouri M, Massah S, Caradec J, Lubik AA, Li N, Truong S (2020). Transient Sox9 Expression Facilitates Resistance to Androgen-Targeted Therapy in Prostate Cancer. Clin Cancer Res.

[35] Liu J, Dong L, Zhu Y, Dong B, Sha J, Zhu HH (2022). Prostate cancer treatment - China's perspective. Cancer Lett.

[36] Devos G, Devlies W, De Meerleer G, Baldewijns M, Gevaert T, Moris L (2021). Neoadjuvant hormonal therapy before radical prostatectomy in high-risk prostate cancer. Nat Rev Urol.

[37] Iglesias-Gato D, Wikstrom P, Tyanova S, Lavallee C, Thysell E, Carlsson J (2016). The Proteome of Primary Prostate Cancer. Eur Urol.

[38] Fraser M, Sabelnykova VY, Yamaguchi TN, Heisler LE, Livingstone J, Huang V (2017). Genomic hallmarks of localized, non-indolent prostate cancer. Nature.

[39] Li J, Xu C, Lee HJ, Ren S, Zi X, Zhang Z (2020). A genomic and epigenomic atlas of prostate cancer in Asian populations. Nature.

[40] Karthaus WR, Hofree M, Choi D, Linton EL, Turkekul M, Bejnood A (2020). Regenerative potential of prostate luminal cells revealed by single-cell analysis. Science.

[41] Joseph DB, Henry GH, Malewska A, Reese JC, Mauck RJ, Gahan JC (2022). 5-Alpha reductase inhibitors induce a prostate luminal to club cell transition in human benign prostatic hyperplasia. J Pathol.

[42] Cheng Q, Butler W, Zhou Y, Zhang H, Tang L, Perkinson K (2022). Pre-existing Castration-resistant Prostate Cancer-like Cells in Primary Prostate Cancer Promote Resistance to Hormonal Therapy. Eur Urol.

[43] Guo W, Li L, He J, Liu Z, Han M, Li F (2020). Single-cell transcriptomics identifies a distinct luminal progenitor cell type in distal prostate invagination tips. Nat Genet.

[44] Kannan A, Clouston D, Frydenberg M, Ilic D, Karim MN, Evans SM (2022). Neuroendocrine cells in prostate cancer correlate with poor outcomes: a systematic review and meta-analysis. BJU Int.

[45] Rodgers JJ, McClure R, Epis MR, Cohen RJ, Leedman PJ, Harvey JM (2019). ETS1 induces transforming growth factor beta signaling and promotes epithelial-to-mesenchymal transition in prostate cancer cells. J Cell Biochem.

[46] Dittmer J (2015). The role of the transcription factor Ets1 in carcinoma. Semin Cancer Biol.

[47] Wang Y, Wang Y, Ci X, Choi SYC, Crea F, Lin D (2021). Molecular events in neuroendocrine prostate cancer development. Nat Rev Urol.

[48] Liu S, Alabi BR, Yin Q, Stoyanova T (2022). Molecular mechanisms underlying the development of neuroendocrine prostate cancer. Semin Cancer Biol.

[49] Heidegger I, Fotakis G, Offermann A, Goveia J, Daum S, Salcher S (2022). Comprehensive characterization of the prostate tumor microenvironment identifies CXCR4/CXCL12 crosstalk as a novel antiangiogenic therapeutic target in prostate cancer. Mol Cancer.

[50] Yu G, Corn PG, Mak CSL, Liang X, Zhang M, Troncoso P (2024). Prostate cancer-induced endothelial-cell-to-osteoblast transition drives immunosuppression in the bone-tumor microenvironment through Wnt pathway-induced M2 macrophage polarization. Proc Natl Acad Sci U S A.

[51] Wang H, Li N, Liu Q, Guo J, Pan Q, Cheng B (2023). Antiandrogen treatment induces stromal cell reprogramming to promote castration resistance in prostate cancer. Cancer Cell.

[52] Zhang Z, Karthaus WR, Lee YS, Gao VR, Wu C, Russo JW (2020). Tumor Microenvironment-Derived NRG1 Promotes Antiandrogen Resistance in Prostate Cancer. Cancer Cell.

[53] Schubert K, Gutknecht D, Koberle M, Anderegg U, Saalbach A (2013). Melanoma cells use Thy-1 (CD90) on endothelial cells for metastasis formation. Am J Pathol.

[54] Chhabra Y, Weeraratna AT (2023). Fibroblasts in cancer: Unity in heterogeneity. Cell.

[55] Bai J, Liu T, Tu B, Yuan M, Shu Z, Fan M (2023). Autophagy loss impedes cancer-associated fibroblast activation via downregulating proline biosynthesis. Autophagy.

[56] Chen Y, Yang S, Tavormina J, Tampe D, Zeisberg M, Wang H (2022). Oncogenic collagen I homotrimers from cancer cells bind to alpha3beta1 integrin and impact tumor microbiome and immunity to promote pancreatic cancer. Cancer Cell.

[57] Li X, Yu X, Bi J, Jiang X, Zhang L, Li Z (2024). Integrating single-cell and spatial transcriptomes reveals COL4A1/2 facilitates the spatial organisation of stromal cells differentiation in breast phyllodes tumours. Clin Transl Med.

[58] Desbois M, Wang Y (2021). Cancer-associated fibroblasts: Key players in shaping the tumor immune microenvironment. Immunol Rev.

[59] Bilusic M, Madan RA, Gulley JL (2017). Immunotherapy of Prostate Cancer: Facts and Hopes. Clin Cancer Res.

[60] Hansen AR, Massard C, Ott PA, Haas NB, Lopez JS, Ejadi S (2018). Pembrolizumab for advanced prostate adenocarcinoma: findings of the KEYNOTE-028 study. Ann Oncol.

[61] Beer TM, Kwon ED, Drake CG, Fizazi K, Logothetis C, Gravis G (2017). Randomized, Double-Blind, Phase III Trial of Ipilimumab Versus Placebo in Asymptomatic or Minimally Symptomatic Patients With Metastatic Chemotherapy-Naive Castration-Resistant Prostate Cancer. J Clin Oncol.

[62] Song H, Weinstein HNW, Allegakoen P, Wadsworth MH 2nd, Xie J, Yang H (2022). Single-cell analysis of human primary prostate cancer reveals the heterogeneity of tumor-associated epithelial cell states. Nat Commun.

[63] Bian X, Wang W, Abudurexiti M, Zhang X, Ma W, Shi G (2024). Integration Analysis of Single-Cell Multi-Omics Reveals Prostate Cancer Heterogeneity. Adv Sci (Weinh).

[64] Obradovic AZ, Dallos MC, Zahurak ML, Partin AW, Schaeffer EM, Ross AE (2020). T-Cell Infiltration and Adaptive Treg Resistance in Response to Androgen Deprivation With or Without Vaccination in Localized Prostate Cancer. Clin Cancer Res.

[65] Shen YC, Ghasemzadeh A, Kochel CM, Nirschl TR, Francica BJ, Lopez-Bujanda ZA (2018). Combining intratumoral Treg depletion with androgen deprivation therapy (ADT): preclinical activity in the Myc-CaP model. Prostate Cancer Prostatic Dis.

[66] Hawley JE, Obradovic AZ, Dallos MC, Lim EA, Runcie K, Ager CR (2023). Anti-PD-1 immunotherapy with androgen deprivation therapy induces robust immune infiltration in metastatic castration-sensitive prostate cancer. Cancer Cell.

[67] Chan JM, Zaidi S, Love JR, Zhao JL, Setty M, Wadosky KM (2022). Lineage plasticity in prostate cancer depends on JAK/STAT inflammatory signaling. Science.

[68] Servant R, Garioni M, Vlajnic T, Blind M, Pueschel H, Muller DC (2021). Prostate cancer patient-derived organoids: detailed outcome from a prospective cohort of 81 clinical specimens. J Pathol.

[69] Linxweiler J, Hammer M, Muhs S, Kohn M, Pryalukhin A, Veith C (2019). Patient-derived, three-dimensional spheroid cultures provide a versatile translational model for the study of organ-confined prostate cancer. J Cancer Res Clin Oncol.

[70] Welti J, Sharp A, Yuan W, Dolling D, Nava Rodrigues D, Figueiredo I (2018). Targeting Bromodomain and Extra-Terminal (BET) Family Proteins in Castration-Resistant Prostate Cancer (CRPC). Clin Cancer Res.

[71] Puca L, Bareja R, Prandi D, Shaw R, Benelli M, Karthaus WR (2018). Patient derived organoids to model rare prostate cancer phenotypes. Nat Commun.

[72] Deng J, Wang ES, Jenkins RW, Li S, Dries R, Yates K (2018). CDK4/6 Inhibition Augments Antitumor Immunity by Enhancing T-cell Activation. Cancer Discov.

[73] Humeniuk MS, Gupta RT, Healy P, McNamara M, Ramalingam S, Harrison M (2018). Platinum sensitivity in metastatic prostate cancer: does histology matter?. Prostate Cancer Prostatic Dis.

[74] Akkus E, Arslan C, Urun Y (2024). Advancements in platinum chemotherapy for metastatic castration-resistant prostate cancer: Insights and perspectives. Cancer Treat Rev.

[75] Cattaneo CM, Dijkstra KK, Fanchi LF, Kelderman S, Kaing S, van Rooij N (2020). Tumor organoid-T-cell coculture systems. Nat Protoc.

[76] Recaldin T, Steinacher L, Gjeta B, Harter MF, Adam L, Kromer K (2024). Human organoids with an autologous tissue-resident immune compartment. Nature.

