# Supplementary material for: Luminal Stem-like Cells in High-Risk/Locally Advanced Prostate Cancer Promote Resistance to Hormonal Therapy
Source: Theranostics. 2025 Aug 30;15(18):9558–79. doi: 10.7150/thno.112496 (PMC12486265; doi:10.7150/thno.112496)
Supplement: Supplementary file 1 — Supplementary figures. [file thnov15p9558s1.pdf]

Supplementary Figures

Luminal stem-like cells in high-risk/locally advanced prostate cancer promote resistance to hormonal therapy

Author: *Guyu Tang[1, #], Jing Liu[2,3, #], Xiaomei Gao[4], Kai Yuan[3], Minfeng Chen[5], Long Wang[1], Lin Qi[5, *], Yi Cai[5, *]*

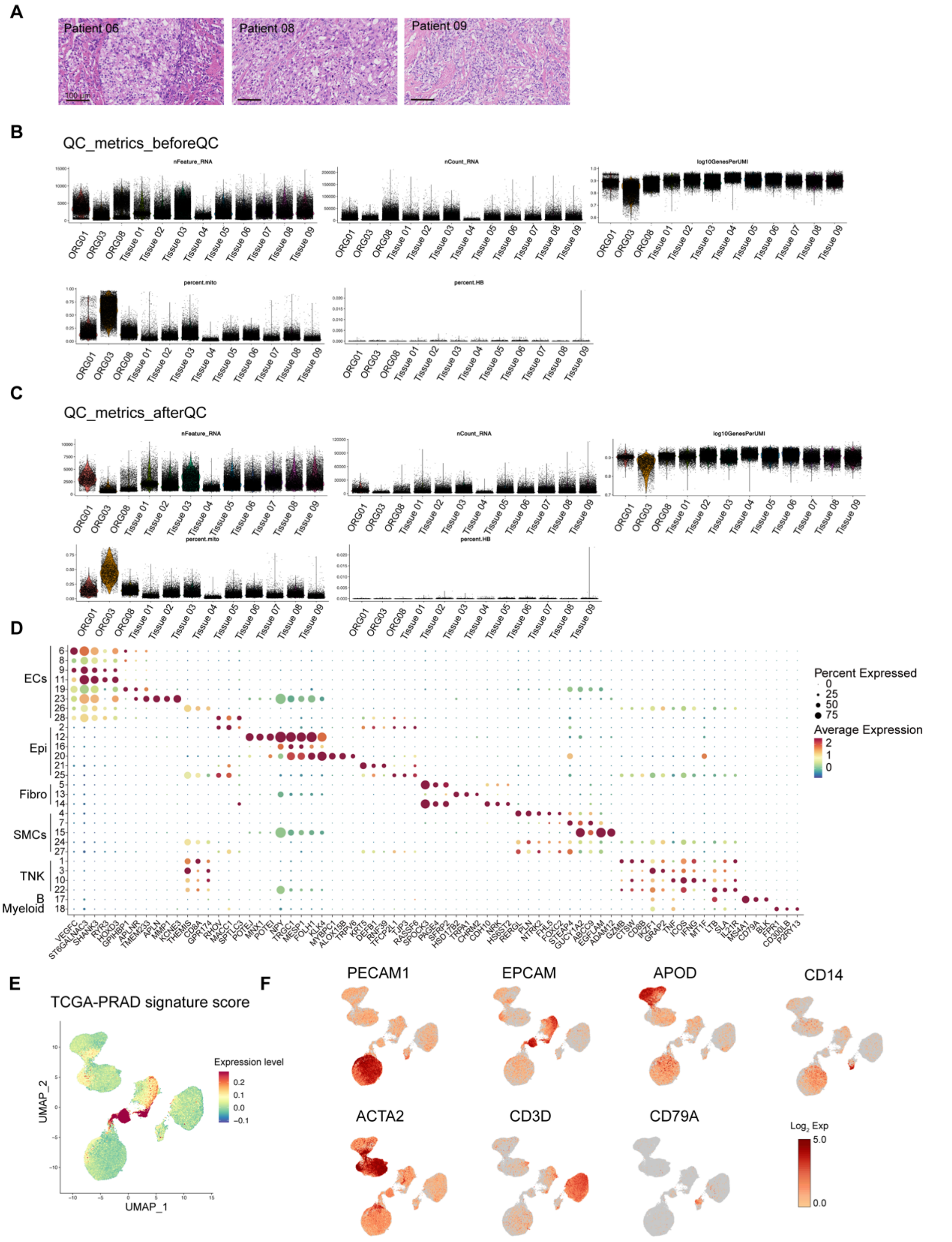

Figure S1 related to figure 1.

(A) H&E staining matched to the scRNA-seq samples (Patient 06, 08, 09).

(B) The data quality for all cells across 9 patients before filtering.

(C) The data quality for all cells across 9 patients after filtering.

(D) Dot plot showing representative marker genes across 28 cell clusters.

(E) UMAP plot showing the TCGA-PRAD signature score of all cell types in 9 tissue

samples.

(F) UMAP plot showing marker genes of 7 cell types. Cells with the highest expression level are colored red.

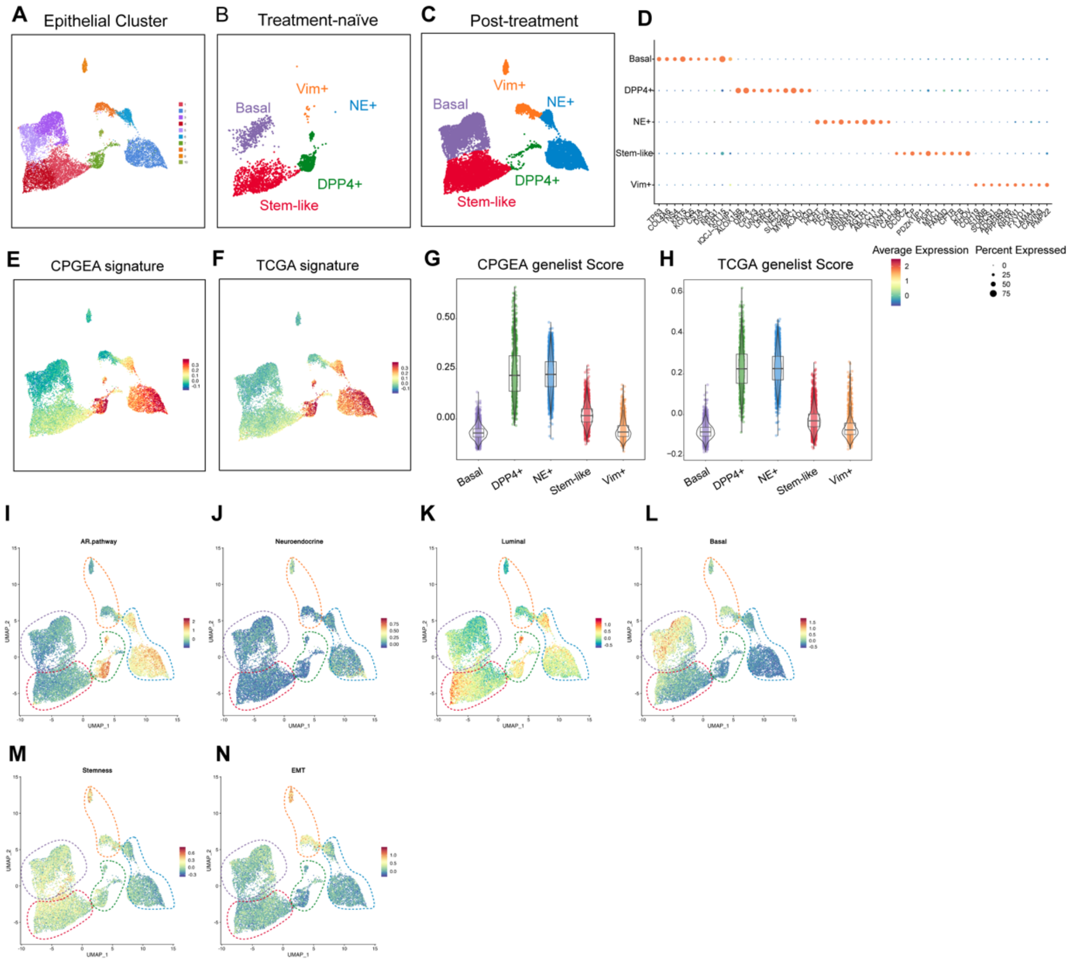

Figure S2 related to figure 2.

(A) UMAP plot showing the clusters of epithelial cells from 9 HRLPC patients, colored by clusters.

(B-C) UMAP plot showing the cells distribution of treatment groups.

(D) Dot plot showing representative top 10 marker genes across 5 epithelial cell subtypes.

(E-F) UMAP plot showing the CPGEA signature score and the TCGA score in epithelial cells.

(G-H) The scatter violin plot showing the CPGEA signature score and TCGA score for

the 5 epithelial cell subtypes.

(I-N) UMAP plot of single-cell transcriptomic profiles colored by AR, luminal, stem-like, NE-like, basal and EMT gene signature score (z score) for each cell (dot).

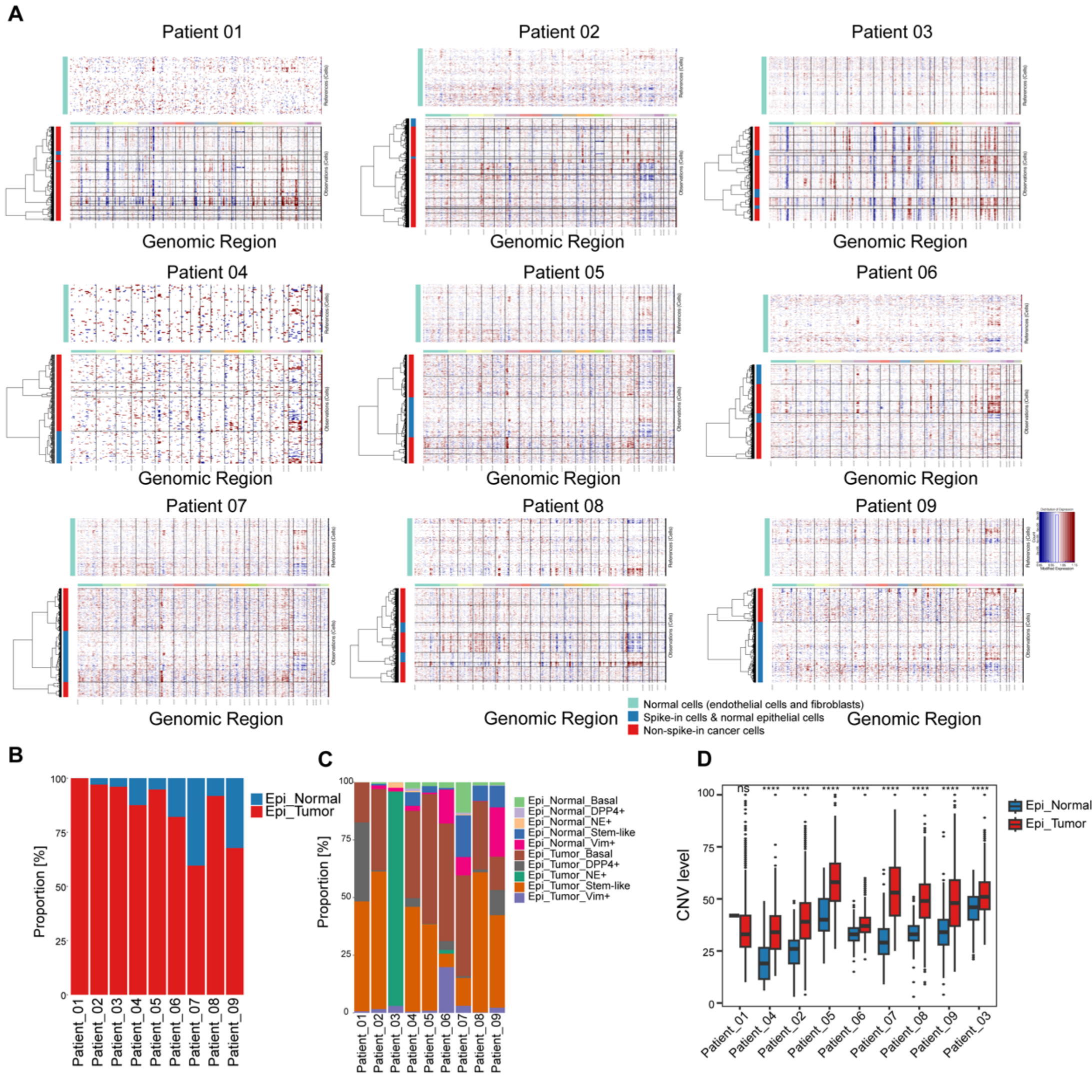

Figure S3 related to figure 2.

(A) The cancer cells and normal epithelial cells are identified by inferCNV in 9 patients.

(B) Bar plot showing the composition of epithelial cells for each patient.

(C) Bar plot showing the composition of epithelial cell subtypes for each patient.

(D) Box plot showing the CNV level of normal epithelial cells and tumor cells in 9 patients.

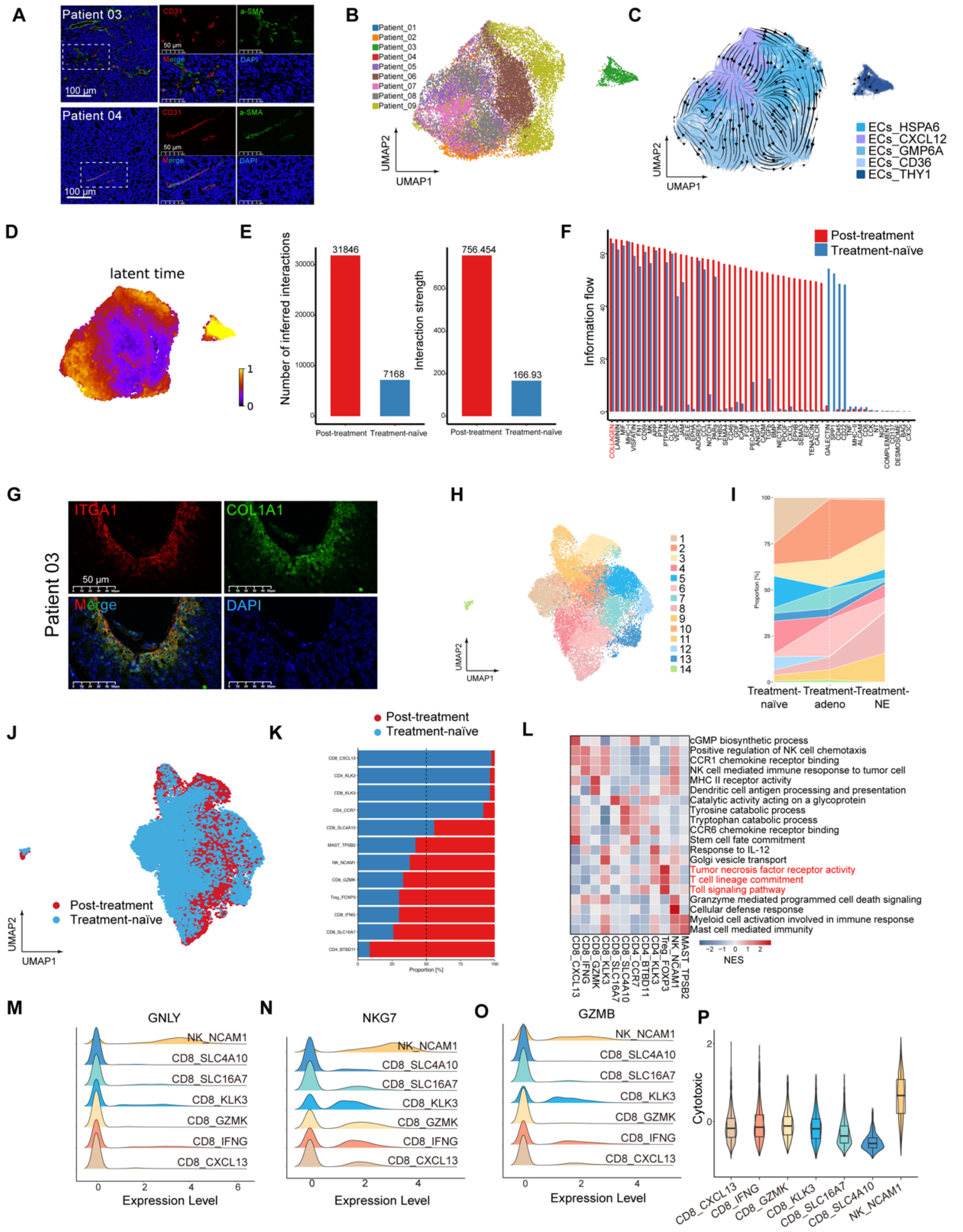

Figure S4 related to figure 4 and 5.

(A) IF staining showing CD31 (red), a-SMA (green) and DAPI (blue) in treatment-naïve (patient 04) and post-treatment (patient 03) samples.

(B-D) UMAP plot showing the distribution of sample names, subtypes, and latent time of ECs from 9 HRLPC patients.

(E) The number of interactions and strength between treatment-naïve and post-

treatment groups.

(F) The bar plot illustrating the signaling pathways between treatment-naïve and post-treatment groups.

(G) IF staining showing ITGA+ (red), COL1A1+ (green) and DAPI (blue) in post-treatment group (patient 03).

(H) UMAP plot showing the clusters of immune cells from 9 HRLPC patients, colored by clusters.

(I) The subtypes proportion changes of three treatment groups of immune cells from 9 HRLPC patients.

(J) UMAP plot showing treatment groups of immune cells from 9 HRLPC patients.

(K) Bar plot displaying the proportion of each immune subtype in the treatment-naïve and post-treatment groups.

(L) The GSVA analysis showing characteristic pathways in each immune subtype.

(M-O) Ridge plots showing the expression levels of GNLY, NKG7 and GZMB in the selected subclusters.

(P) Violin plots depicting the cytotoxicity scores in the selected subtypes. Cytotoxic genes are provided in Supplementary Table S5.

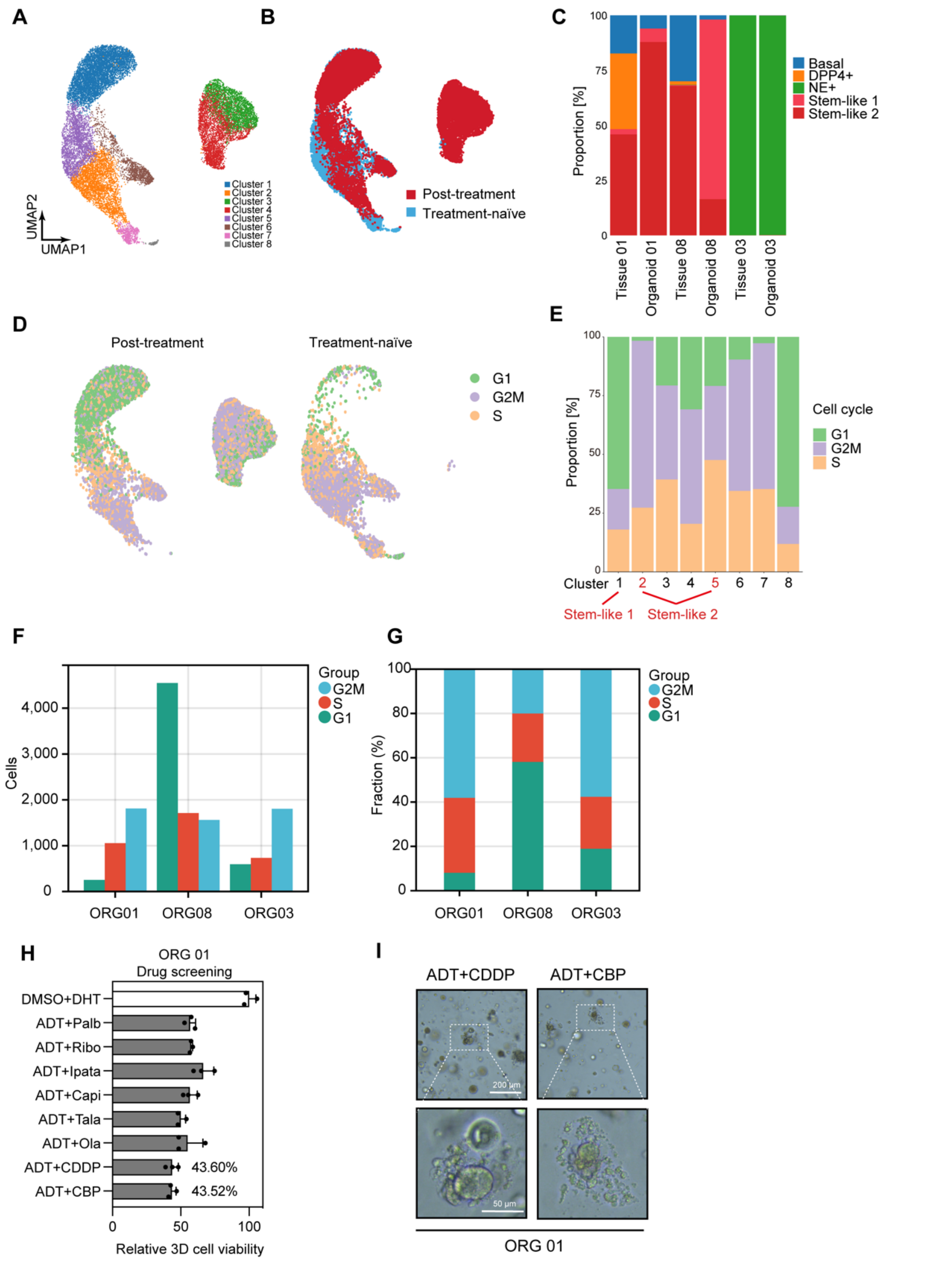

Figure S5 related to figure 7.

(A-B) UMAP plot showing the clusters and treatment groups of epithelial cells from 3 pairs of tissues and PDOs.

(C) The stacked bar chart representing relative abundance of 3 pairs of tissues and PDOs in epithelial subtypes.

(D) UMAP plot showing the cell cycle states of epithelial cells from treatment-naïve and post-treatment groups.

(E) The stacked bar chart representing relative abundance of the cell cycle states in epithelial cell clusters.

(F-G) The stacked bar chart representing cell number (F) and relative abundance (G) of 3 PDOs in different cell cycle phases.

(H-I) Bar plot and organoids bright-field images showing the relative 3D cell viability after treatment in treatment-adeno (patient 01).
